# Tunable Synthesis of 2D Bismuth Oxyhydroxide and Oxysulfide from Solid–Liquid Interfacial Reaction for High Performance Optoelectronic Gas Sensing

**DOI:** 10.1002/smll.202411522

**Published:** 2025-03-10

**Authors:** Tao Tang, Zhong Li, Li Zhou, Pu Zhang, Yin Fen Cheng, Yi Liang, Jing Hao Zhuang, Xin Yi Hu, Qi Jie Ma, Bao Yue Zhang, Azmira Jannat, Jian Zhen Ou

**Affiliations:** ^1^ Key Laboratory of Advanced Technologies of Materials Ministry of Education School of Materials Science and Engineering Southwest Jiaotong University Chengdu 610031 China; ^2^ Jiangsu Key Laboratory of Advanced Structural Materials and Application Technology Nanjing Institute of Technology Nanjing 211167 China; ^3^ Research Institute of Natural Gas Technology Petro China Southwest Oil and Gas field Company Chengdu 610213 China; ^4^ Institute of Advanced Study Chengdu University Chengdu 610106 China; ^5^ School of Engineering RMIT University Melbourne Victoria 3000 Australia

**Keywords:** 2D material, gas sensor, oxysulfide, solid–liquid interfacial reaction, tunable band structure

## Abstract

The self‐limiting Cabrera–Mott oxidation reaction on metal surfaces provides an effective pathway for synthesizing atomically thin 2D metal oxide. Inspired by this reaction, it is proposed that solid bismuth metals can react with dissolved oxygen and water molecules in an aqueous environment, forming an ultrathin oxyhydroxide layer on their surfaces. The lattice mismatch between the surface oxyhydroxide layer and the underlying pure metal enables the mechanical exfoliation of detached 2D oxyhydroxide nanosheets. Moreover, the sulfurization interaction between the oxyhydroxide and dissolved H_2_S expands the applicability of solid–liquid interfacial reactions for realizing 2D bismuth oxysulfide, effectively tuning their electronic bandgap energy, work function, and band position. Given its good photoresponse from blue to UV light, the optoelectronic gas sensing performances of bismuth oxysulfide are investigated. Under purple light irradiation, the Bi_2_O_1.12_S_1.88_‐based gas sensor exhibits an excellent optoelectronic response factor of 48.5% toward 10 ppb NO_2_, which is the lowest detection limit for reported bismuth compounds‐based gas sensor so far. This work provides a novel and facile synthesis approach for 2D metal oxyhydroxide and oxysulfide and simultaneously demonstrates the substantial potential of bismuth oxysulfide in high‐performance optoelectronic gas sensing.

## Graphical abstract

The solid–liquid interfacial reaction of bismuth metal in different aqueous environments enables the tunable synthesis of 2D bismuth oxyhydroxide and oxysulfide. The introduction of S atoms in bismuth oxysulfide induces a redshift optical adsorption, thus achieving photodetection in the visible light range. Modulation of energy band structure by chemical composition makes Bi_2_O_1.12_S_1.88_‐based sensor present excellent optoelectronic gas response toward ultra‐low NO_2_ concentration.

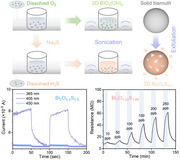

## 1 Introduction

Research on metal oxidation indicates that an ultrathin self‐limiting oxide layer rapidly forms on metal surfaces when exposed to an oxygen atmosphere according to the Cabrera–Mott oxidation model.^[^ ^1^, ^2^ ^]^ Recently, the formation of the self‐limiting atomically thin oxide at the interface between metal and air has gained significant attention for synthesizing high‐quality 2D metal oxides.^[^ ^3^, ^4^, ^5^ ^]^ Following Cabrera–Mott oxidation, an ultrathin oxide layer grows on the surface of liquid metal exposed to oxygen atmosphere. Due to its soft liquid state, the surface oxide layer is weakly attached and can easily be exfoliated onto desired substrates, which successfully achieves the synthesis of 2D Ga_2_O_3_, In_2_O_3_, SnO, Bi_2_O_3_, HfO_2_, Al_2_O_3_, Gd_2_O_3_, TeO_2_, and Sb_2_O_3_.^[^ ^6^, ^7^, ^8^, ^9^, ^10^, ^11^ ^]^ Moreover, a layered planar hexagonal phase of oxide is discovered on the solid transition metals, post‐transition metals, lanthanides, and metalloids, derived from strictly controlled oxidation at the metal‐gas interface. The highly crystalline monolayers, without the support of ionic dopants or vacancies, can easily be mechanically exfoliated by stamping them onto substrates, thus 2D TiO_2_, MnO, Fe_2_O_3_, CoO, Ni_2_O_3_, and Cu_2_O are obtained.^[^ ^12^ ^]^ Both approaches demonstrate that metal platforms are an effective pathway to realize the synthesis of 2D metal oxides.

**Figure 1:**
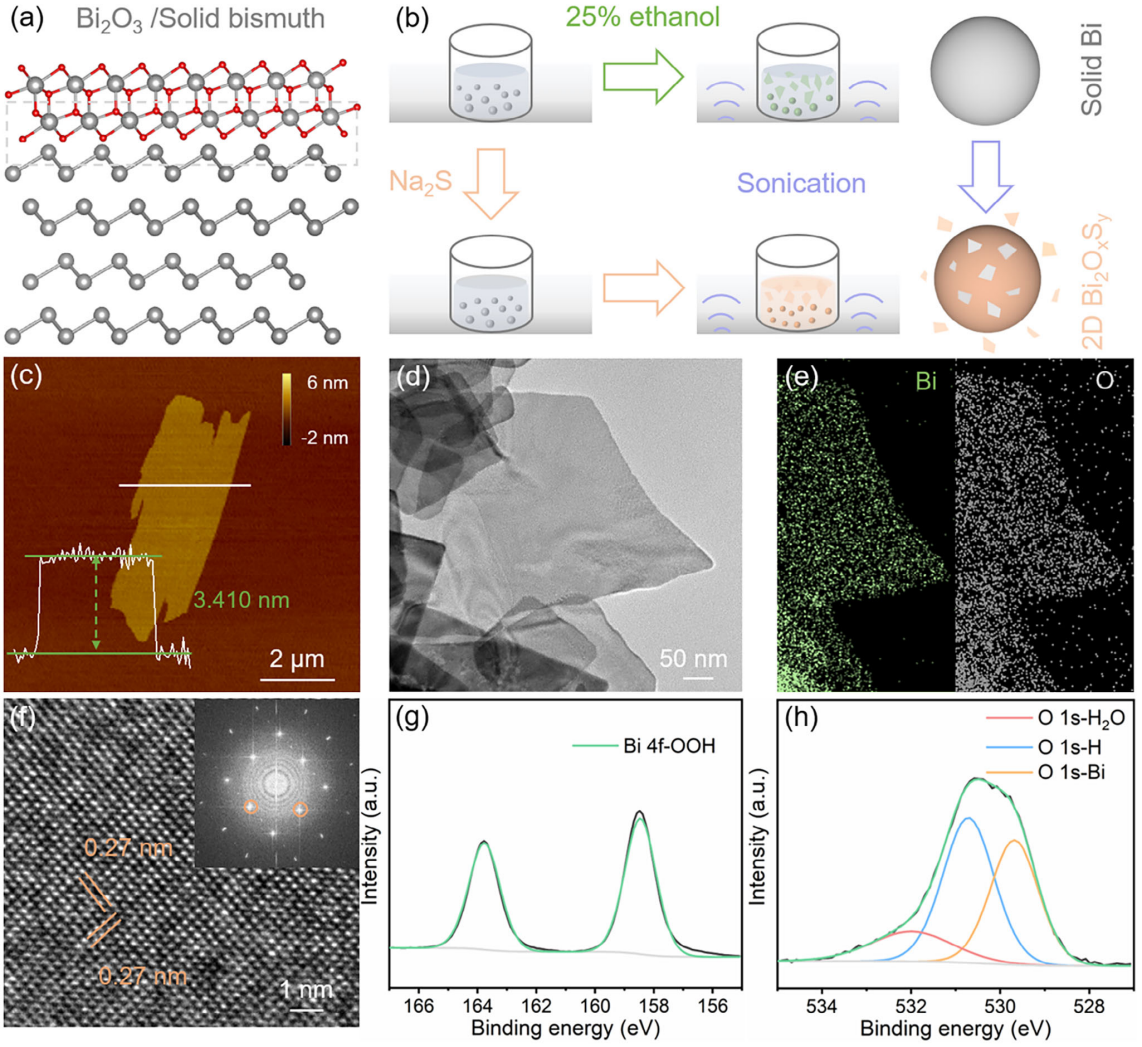
a) The lattice mismatch in the crystal structure of bismuth metal after surface oxidation. b) Schematic illustration of the preparation of 2D bismuth oxyhydroxide and oxysulfide. c) The AFM images of bismuth oxyhydroxide nanosheets. d,e) TEM image and corresponding ESD mapping. f) The HRTEM image with the corresponding FFT pattern. g) Bi 4f region and h) O 1s region of XPS spectra.

Inspired by the Cabrera–Mott oxidation process in metals, we propose a strategy of ultrasonicating solid metal in an aqueous environment to synthesize 2D metal oxides in high yield. Compared with the synthesis of 2D metal oxide based on a liquid metal platform, this method can break the high dependence on the liquid state that arises from the need for a soft and high active surface.^[^ ^3^, ^7^ ^]^ Furthermore, unlike the need for artificial control of the oxidation environment in a metal‐gas system, the dissolved oxygen in the aqueous environment naturally provides an extremely low oxygen environment conducive to the growth of an ultrathin surface oxide layer in this solid–liquid system.^[^ ^12^ ^]^ Meanwhile, ultrasound serves as an alternative to manual mechanical force, continuously promoting the exfoliation of the surface oxide layer, thereby providing the possibility for synthesizing 2D metal oxides in high yield.^[^ ^13^, ^14^ ^]^ Bismuth metal (Bi), a typical group VA elemental material with a melting point of 271.4 °C,^[^ ^15^, ^16^ ^]^ reacts with dissolved oxygen during sonication in an aqueous environment, forming an ultrathin bismuth oxide layer on its surface. The significant difference in lattice parameters between the surface oxide layer (a = 0.4546 nm, b = 0.4546 nm, c = 11.8020 nm) and inner pure bismuth (a = 0.7740 nm, b = 0.7740 nm, c = 0.5644 nm) may lead to the lattice mismatch at their interface (**Figure** **1**a),^[^ ^17^, ^18^ ^]^ weakening the interaction between the oxide layer and bismuth. Benefiting from this lattice mismatch, it is possible to exfoliate the oxide layer from the bismuth surface by breaking their interactions under ultrasonic force, ultimately forming suspended 2D metal oxide nanosheets. While the water molecule also possesses the ability to react with bismuth metal in an aqueous environment.^[^ ^19^, ^20^ ^]^ Consequently, the reaction between bismuth metal, oxygen, and water molecules leads to the growth of bismuth oxyhydroxide on the bismuth metal surface.

Metal oxysulfides, another derivative of 2D metal oxides, have recently attracted significant interest in various fields such as optoelectronics, catalysis, and sensors. The oxygen and sulfur atoms in metal oxysulfide simultaneously bond with metal atoms, forming the alternating stack of oxide and sulfide.^[^ ^21^, ^22^ ^]^ Thus, the coexistence of oxygen and sulfur atoms is able to achieve more flexible energy band structure modulation. Moreover, the orbital hybridization between oxygen and sulfur atoms can passivate surface sulfur atoms, thereby improving material stability.^[^ ^23^, ^24^ ^]^ Among various metal oxysulfides, bismuth oxysulfide exhibits efficient charge dissociation, high charge carrier transport, and long carrier lifetime, which is considered to be the wide application prospects in next generation electronic and optoelectronic devices.^[^ ^25^, ^26^ ^]^ However, the application of bismuth oxysulfide is limited due to the lack of a facile and scalable synthesis method. Notably, the solid–liquid system offers a well‐controlled and less toxic platform for synthesizing 2D metal oxysulfide through wet chemical reactions. Previous research indicates that hydroxide, as an active catalyst for removing H_2_S, can trigger the dissociation of dissolved H_2_S into HS^−^ and S^2−^by interacting with it.^[^ ^27^, ^28^, ^29^ ^]^ Therefore, the sulfurization interaction between oxyhydroxide and dissolved H_2_S is a viable pathway for converting oxyhydroxide to oxysulfide, which is perfectly compatible with the solid–liquid system.

Herein, we investigate the synthesis of 2D bismuth oxyhydroxide and oxysulfide in a solid–liquid system. When solid bismuth is sonicated in a pure aqueous environment, an ultrathin oxyhydroxide layer forms on the bismuth surface through reactions with oxygen and water molecules. The introduction of dissolved H_2_S facilitates the conversion of bismuth oxyhydroxide to oxysulfide. Importantly, the lattice mismatch between the surface layers and the inner pure bismuth allows for the detachment of 2D bismuth oxyhydroxide and oxysulfide nanosheets. Regulating the oxygen–sulfur ratio in 2D bismuth oxysulfide effectively tunes their electronic structures, including bandgap, work function, and energy band position, greatly enhancing their application in optoelectronic gas detection. Bismuth oxysulfide‐based photodetectors exhibit a broad photoresponse from blue to UV light. Given these optoelectronic properties, the gas sensing performance of bismuth oxysulfide‐based senor has been investigated at room temperature under light excitation.

## 2 Results and Discussion

The synthesis of ultrathin 2D bismuth oxyhydroxide and oxysulfide is achieved via solid–liquid reaction in different aqueous environments as illustrated in Figure 1b. As sonicated in an aqueous environment, the bismuth metal is able to react with dissolved oxygen and water molecules, thus prompting ultrathin self‐limiting oxyhydroxide layers to grow on its surface as described in Equation 1. The different lattice parameters between surface oxyhydroxide layers and bismuth metal will lead to the lattice mismatch at their interface, which can reduce the interaction between the surface layers and inner bismuth. Hence, surface oxyhydroxide layers can be exfoliated from the bismuth surface through breaking the weakened interaction, finally forming a suspended 2D nanosheet as shown in Figure S1 (Supporting Information). Bismuth particles exhibit darkened luster and uneven surface morphologies after being sonicated in an aqueous environment due to the growth and exfoliation of surface bismuth oxyhydroxide (Figure S2, Supporting Information). It is found that the productivity of nanosheets is related to the ratio of ethanol to water, which reaches the maximum in the 25%v/v ethanol (Figure S3, Supporting Information). This may be due to that the surface tension of 25%v/v ethanol matches well with the interaction between the oxyhydroxide layers and bismuth particles, which is similar to the liquid‐phase exfoliation of layered van‐der‐Waals materials.^[^ ^13^, ^30^ ^]^ Moreover, the temperature effect during the ultrasonic process on the synthesis yield of bismuth oxyhydroxide has been studied. As shown in Figure S4 (Supporting Information), the dispersion after sonicated bismuth particles in 86, 73, and 57 °C exhibit different colors, in which the darkest color in 86 °C demonstrates the highest concentration of bismuth oxyhydroxide nanosheets according to colorimetry. Consequently, the increasing temperature of the ultrasonic process can effectively improve the synthesis yield due to and the high temperature is able to promote the growth of the surface oxyhydroxide layer. However, excessively high temperatures may cause the ultrasound machine to overheat, so the temperature during the ultrasonic process is chosen as 86 °C for the synthesis of bismuth oxyhydroxide and oxysulfide. After that, the Na_2_S is introduced in a pure aqueous environment to provide a hydrogen sulfide (H_2_S) atmosphere after reacting with water (Equation 2).^[^ ^31^, ^32^ ^]^ The bismuth oxysulfide layer is then formed on the surface of bismuth particles under the sulfurization effect of dissolved H_2_S toward oxyhydroxide layers (Equation 3). To further demonstrate the sulfurization interaction between dissolved H_2_S and bismuth oxyhydroxide, the H_2_S gas was directly injected into the dispersion of bismuth oxyhydroxide. As shown in Figure S5 (Supporting Information), there is a significant color change after injected H_2_S, which indicates the sulfurization interaction between bismuth oxyhydroxide and dissolved H_2_S. Finally, bismuth oxysulfide layers will also detach from the surface, forming suspended 2D oxysulfide nanosheets due to the existence of lattice mismatch between bismuth metal and surface bismuth oxysulfide layers as shown in Figure S1 (Supporting Information). Bismuth particles after being sonicated in Na_2_S solution present brown color and increased surface roughness, possibly due to the exfoliation of surface bismuth oxysulfide, and the surface corrosion of bismuth particles by the alkaline Na_2_S solution (Figure S2, Supporting Information). Compared with the synthesis of oxysulfide via a liquid metal platform, hydrothermal method, and high‐temperature solid‐state reaction in special gas atmosphere, this method is conducted at a low‐temperature sonication environment with facile operation.^[^ ^21^, ^23^, ^33^, ^34^ ^]^

(1) $$\begin{array}{c} \mathrm{Bi}+O_{2}+H_{2}O\to {\mathrm{BiO}}_{x} \end{array}{\left(\mathrm{OH}\right)}_{y}$$

(2) $$\begin{array}{c} {\mathrm{Na}}_{2}S+2H_{2}O\to H_{2}S+2\mathrm{NaOH} \end{array}$$

(3) $$\begin{array}{c} {\mathrm{BiO}}_{x}{\left(\mathrm{OH}\right)}_{y}+H_{2}S\;\to \;{\mathrm{Bi}}_{2}O_{x}S_{3-x}+{\mathrm{yH}}_{2}O \end{array}$$

The morphology of the synthesized bismuth oxyhydroxide nanosheets is investigated using atomic force microscopy (AFM) and scanning electron microscopy (SEM). AFM image in Figure 1c indicates that the nominal thickness of 2D bismuth oxyhydroxide nanosheets is ≈3.410 nm. A statistical analysis shows that the thickness distribution has a peak centered at ≈3.506 nm (Figure S6, Supporting Information). The SEM image in Figure S7a (Supporting Information) shows that the lateral dimensions of bismuth oxyhydroxide nanosheets present a small dimension, which is mainly ranging at a few micrometers according to its statistical analysis (Figure S7b, Supporting Information). The transmission electron microscopy (TEM) image and energy dispersive X‐ray (EDX) mapping results (Figure 1d,e) confirm that in the bismuth oxyhydroxide nanosheet, the Bi and O elements are uniformly distributed. The high‐resolution TEM image together with the corresponding fast Fourier transform (FFT) pattern in Figure 1f indicate that the bismuth oxyhydroxide nanosheets are high crystallinity and have well‐ordered lattice fringes. The lattice spacing of 0.27 nm and FFT pattern are identical to the known compound Bi_2_O_2_(OH)(NO_3_), which not only contains a layer structure constructed by [Bi_2_O_2_(OH)]^+^ layers and interleaved NO_3_^−^ slices along the *z*‐axis, but also crystallizes in a noncentrosymmetric polar orthorhombic crystal structure.^[^ ^35^, ^36^, ^37^ ^]^ Therefore, we infer that the bismuth oxyhydroxide has a similar crystal structure with Bi_2_O_2_(OH)(NO_3_), while the interleaved anions are absent. X‐ray photoelectron spectroscopy (XPS) has been used to study the surface state and chemical composition of bismuth oxyhydroxide. The Bi 4f spectra in Figure 1g show that the peaks centered at 158.5 and 163.8 eV are attributed to the Bi 4f_7/2_ and Bi 4f_5/2_ of Bi^3+^ in BiO_x_(OH)_y_.^[^ ^38^, ^39^ ^]^ Figure 1h shows the O 1s peaks at 529.7, 530.9, and 532.0 eV, corresponding to the O atoms in Bi─O bonds, H─O bonds, and surface adsorbed H_2_O, respectively.^[^ ^36^, ^37^, ^39^ ^]^ Furthermore, Raman spectroscopy and Fourier transform infrared (FTIR) are further engaged to reveal the bonding information in bismuth oxyhydroxide. From Raman spectra in Figure S8a (Supporting Information), the vibration peaks at 272.86, 329.49, 531.24, and 615.91 cm^−1^ are all characteristic modes of Bi─O bonds.^[^ ^40^, ^41^ ^]^ The FTIR spectra in Figure S8b (Supporting Information) more directly prove the existence of O─H bonds in bismuth oxyhydroxide. The peaks located at 470.06, 597.79, 831.17, and 1084.76 cm^−1^ are related to the stretching vibration modes of Bi─O bonds.^[^ ^42^, ^43^, ^44^ ^]^ The peak centered at 1391.87 cm^−1^ corresponds to Bi─OH bonds.^[^ ^45^ ^]^ Another two peaks at 1632.45 and 3454.36 cm^−1^ are the modes of H─O─H vibration in adsorbed water molecules.^[^ ^46^ ^]^

**Figure 2:**
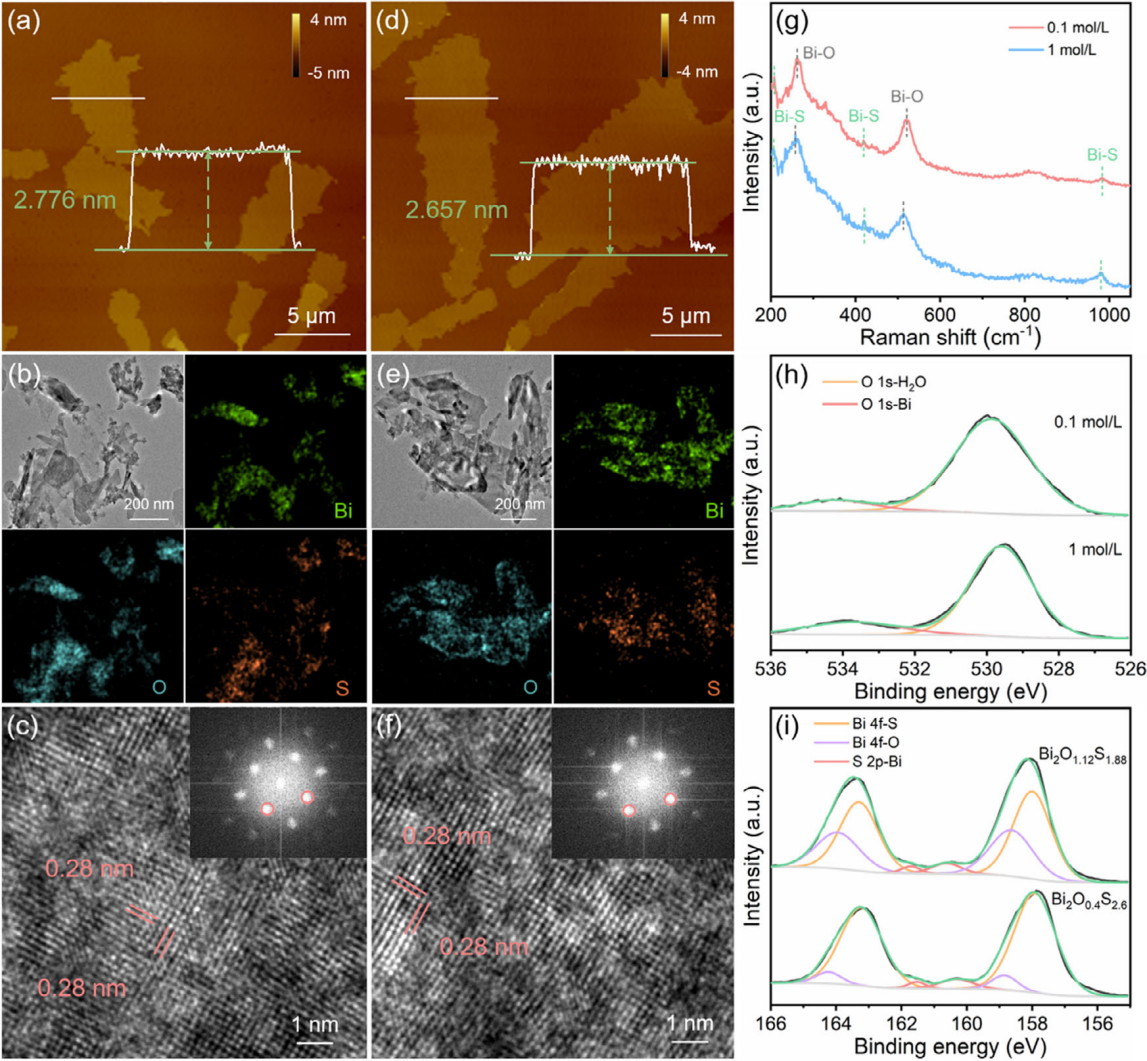
Morphological and chemical composition characterizations of bismuth oxysulfide. a,d) The AFM images. b,e) The TEM images with corresponding EDS mapping. c,f) HRTEM images with corresponding FFT patterns. g) Raman spectra. h) O 1s region and i) Bi 4f region of XPS spectra.

When the Na_2_S is introduced in a solid–liquid system, the conversion of bismuth oxyhydroxide to bismuth oxysulfide is achieved (Figure 1b), in which two types of bismuth oxysulfide are obtained in different concentrations. Similar to the bismuth oxyhydroxide, both types of bismuth oxysulfide present a 2D nanosheet morphology as revealed by AFM and TEM. The bismuth oxysulfide synthesized in 0.1 mol L^−1^ Na_2_S solution exhibits a thickness of 2.776 nm as shown in **Figure** **2**a. The corresponding statistical analyses in Figures S9 and S10 (Supporting Information) indicate that their thickness and lateral dimension distributions of bismuth oxysulfide nanosheets have a peak centered at 2.848 nm and 13.696 µm, respectively. As shown in Figure 2d, the bismuth oxysulfide synthesized in 1 mol L^−1^ Na_2_S solution presents a thickness of 2.657 nm. The statistical analyses in Figures S11 and S12 (Supporting Information) indicate that their thickness and lateral dimensions are mostly 2.793 nm and 23.344 µm. The thinner thickness and larger lateral dimension of bismuth oxysulfide than bismuth oxyhydroxide could be attributed to that the intercalation of Na^+^ into the interface space between oxysulfide layers and bismuth surface is able to reduce their interactions, thus further facilitating the exfoliation of surface layers.^[^ ^47^ ^]^ TEM images with corresponding EDS mapping in Figure 2b,e indicate the existence and uniform distribution of Bi, O, and S elements in bismuth oxysulfide, confirming that the S atoms have been introduced successfully. HRTEM images and the corresponding FFT patterns in Figure 2c,f reveal that the crystalline structure is almost identical with bismuth oxyhydroxide. The lattice spacing slightly increases from 0.27 to 0.28 nm after the introduction of S atoms, which could be due to that the radius of S atoms (1.7 Å) is larger than that of O atoms (1.29 Å) and OH^−^ ions (1.33 Å).^[^ ^48^, ^49^ ^]^ As shown in Raman spectra of two types of bismuth oxysulfide in Figure 2g, the peaks located at 262.21 and 531.32 cm^−1^ are attributed to the Bi─O vibrational modes.^[^ ^40^, ^41^ ^]^ The peaks appeared at 207.17, 418.60, and 982.36 cm^−1^ correspond to the vibration of Bi─S bonds,^[^ ^50^, ^51^ ^]^ which further confirms the successful introduction of S atoms. In addition, the XPS spectra are carried out to reveal the chemical composition of bismuth oxysulfide. The O 1s region in Figure 2h shows two main peaks at 529.58 and 533.68 eV, corresponding to the O atoms in Bi─O bonds and surface adsorbed H_2_O, respectively,^[^ ^36^, ^37^, ^39^ ^]^ in which the absence of characteristic peak for H─O bonds in O 1s region confirms that the conversion of oxyhydroxide into oxysulfide is achieved. As shown in Figure 2i, the peaks located at 160.52 and 161.80 eV ascribe to the S 2p_3/2_ and S 2p_1/2_ of S─Bi bonds in bismuth oxysulfide. The peaks at 158.04 and 163.32 eV are attributed to the Bi 4f_7/2_ and Bi 4f_5/2_ in Bi─S bonds. The peaks at 158.66 and 164.0 eV correspond to the Bi 4f_7/2_ and Bi 4f_5/2_ in Bi─O bonds.^[^ ^52^, ^53^ ^]^ The peak area ratio of Bi─S to Bi─O is 1:0.6 and 1:0.15, which proves that the estimated bismuth oxysulfide compounds synthesized in 0.1 and 1mol L^−1^ Na_2_S is Bi_2_O_1.12_S_1.88_ and Bi_2_O_0.4_S_2.6_, respectively.

**Figure 3:**
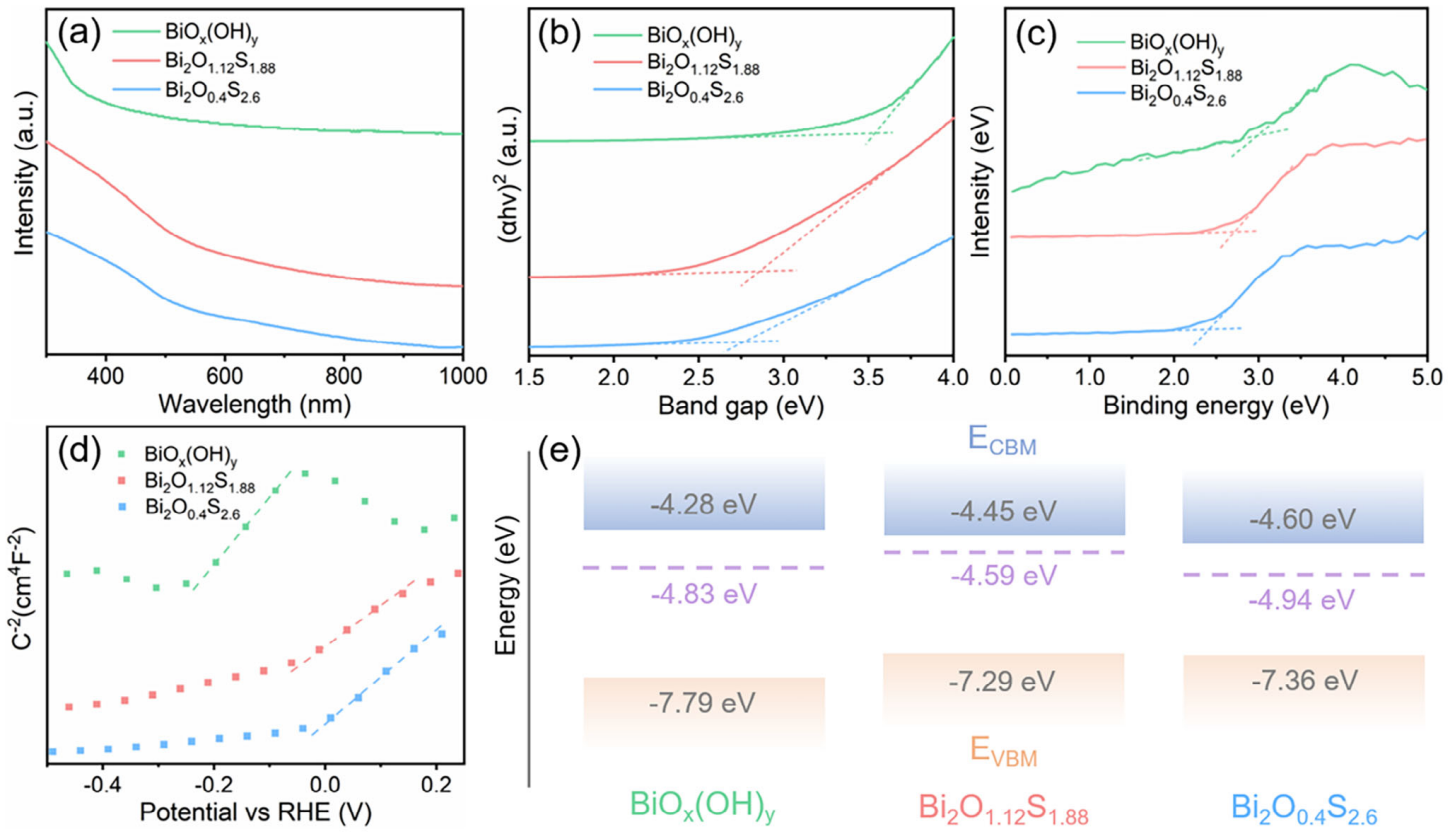
Optoelectronic properties of bismuth oxyhydroxide and oxysulfide. a) UV–vis–NIR absorption spectra. b) corresponding Tauc plot. c) XPS valence band spectra. d) Mott–Schottky plot. e) schematic diagram of the energy band structure.

The optical properties of bismuth oxyhydroxide and oxysulfide have been studied via UV–vis–NIR absorption spectra. As shown in **Figure** **3**a, the introduction of S atoms in bismuth oxyhydroxide induces a redshift of the adsorption band edge from ≈370 to ≈550 nm, which means that the bismuth oxysulfide has enhanced absorption in the visible region. The direct bandgaps calculated by the Tauc plot method in Figure 3b are 3.51, 2.84, and 2.76 eV for the BiO_x_(OH)_y_, Bi_2_O_1.22_S_1.88_, and Bi_2_O_0.4_S_2.6_, which proves that the bandgap of bismuth oxyhydroxide and oxysulfide can be modulated by the introduction and controlling the concentration of S atoms. In order to further explore the influence of S atoms on the electronic band structure, the VB‐XPS and Mott–Schottky measurements have been conducted. The energy difference between the valence band maximum (VBM) and the Fermi energy level (E_Femi_) is obtained by calculating the onset energy from the valence band spectra in Figure 3c. The difference values are 2.96, 2.70, and 2.42 eV for BiO_x_(OH)_y_, and Bi_2_O_1.22_S_1.88_ and Bi_2_O_0.4_S_2.6_, respectively. The positive slopes of Mott–Schottky plots in Figure 3d indicate that both bismuth oxyhydroxide and oxysulfide exhibit *n*‐type semiconductor behavior. According to the Mott–Schottky plots, the flat band potential of BiO_x_(OH)_y_, Bi_2_O_1.22_S_1.88_, and Bi_2_O_0.4_S_2.6_ are calculated to be −0.40, −0.23, and −0.08 eV, respectively. The conduction band minimum (CBM) can be obtained via the formula of *E_CBM_*  =  *E*_*Ag*/*AgCl*_ – *E*_*Flat* *band*_, in which *E*_*Ag*/*AgCl*_ is the reference electrode potential with respect to vacuum.^[^ ^54^, ^55^ ^]^ As a result, the CBM of BiO_x_(OH)_y_, Bi_2_O_1.22_S_1.88_, and Bi_2_O_0.4_S_2.6_ are located at −4.28, −4.45, and −4.60 eV. Finally, the energy band structures of bismuth oxyhydroxide and oxysulfide are established based on the above results as shown in Figure 3e. It is found that the changes in bandgap are derived from the reduced energy difference between the Femi level and the VBM as well as CBM, which could be due to the hybridization between S 3p orbitals and Bi 4f orbitals will introduce a new unoccupied band in bismuth oxysulfide.^[^ ^56^, ^57^, ^58^ ^]^

**Figure 4:**
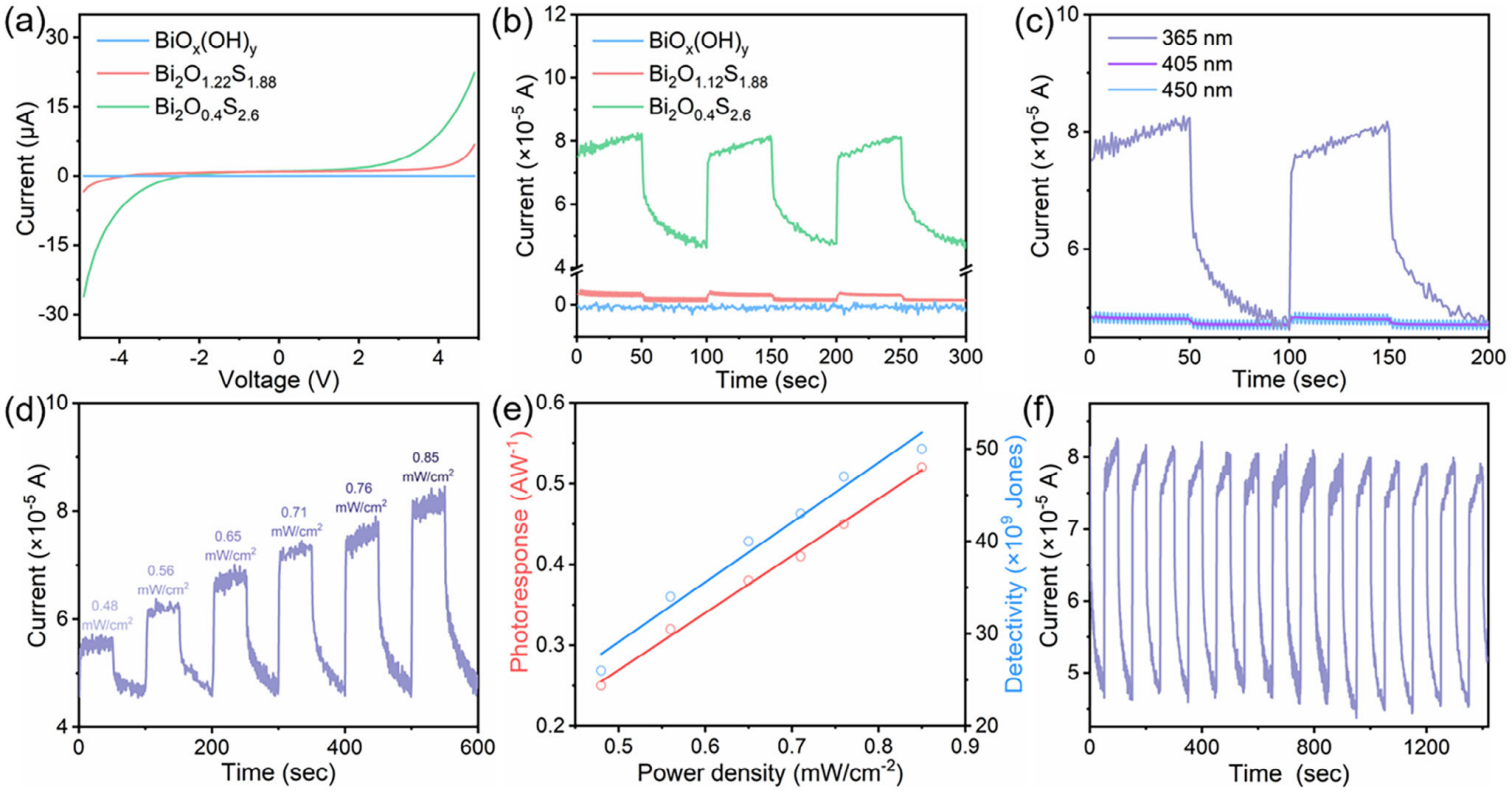
Photoresponse of bismuth oxyhydroxide and oxysulfide. a) *I*–*V* characteristic curves. b) Photocurrent response toward UV light. c) Dynamic photocurrent response of Bi_2_O_0.4_S_2.6_ for UV, purple, and blue light. d) Dynamic photocurrent response of Bi_2_O_0.4_S_2.6_ at UV light with the power densities of 0.48, 0.56, 0.65, 0.71, 0.76, and 0.85 mW cm^−2^. e) Photoresponse and detectivity as a function of the power density. f) The repeatability of Bi_2_O_0.4_S_2.6_ photodetector at the illumination wavelength of UV light.

Based on the above optical properties of bismuth oxyhydroxide and oxysulfide, the viability for photodetection application has been studied. The *I*–*V* curves in **Figure** **4**a reveal the electrical properties of bismuth oxyhydroxide and oxysulfide. It is found that bismuth oxysulfide presents a higher current (µA) compared with bismuth oxyhydroxide (nA) at the same bias voltage. The higher current presents advantages such as higher signal strength, stronger anti‐interference ability, wider dynamic range, and better circuit compatibility, which is more appropriate for the photodetector and resistive type gas sensor. The photoresponse measurements of bismuth oxyhydroxide and oxysulfide are conducted under UV (365 nm), purple (405 nm), and blue (450 nm) light illumination, respectively. Figure 4b illustrates the photocurrent response of bismuth oxyhydroxide and oxysulfide toward UV light. It is found that Bi_2_O_0.4_S_2.6_ exhibits the highest photocurrent, which may attribute to that the narrow energy bandgap could produce more photoexcited carriers under the same light illumination. As shown in Figure 4c, the photodetector of Bi_2_O_0.4_S_2.6_ exhibits a photoresponse region from blue to UV light. Upon the increase of wavelength, the responsivity is decreased from 0.52 to 0.012 A W^−1^. Similarly, the photodetectivity also decreased from 5.0 × 10^9^ to 1.3 × 10^8^ Jones. Both responsivity and detevtivity are decreased as the wavelength increases, which may be due to the higher excitation energy can produce more photogenerated carriers in Bi_2_O_0.4_S_2.6_.^[^ ^59^ ^]^ The photoresponse (*R*) is defined as the photocurrent generated per unit power of incident light on the selective area of a photodevice. The *R*‐value can be calculated according to the equation of *R*  =  *I_ph_*/*PS*, where *I_ph_* is generated photocurrent

(4) $$I_{\mathrm{ph}}=I_{\mathrm{illumination}}\;-\;I_{\mathrm{dark}}$$

*P* is the incident light intensity, *S* is the effective area, respectively. The photodetectivity (D^*^) is defined as

(5) $$D^{*}=RS^{1/2}/{\left(2qI_{\mathrm{dark}}\right)}^{1/2}$$

in which *R*, *S*, *q*, and *I_dark_* are the photoresponsivity, effective area, electronic charge, and dark current, respectively.^[^ ^60^ ^]^ Figure 4d reveals that the photocurrent increases with light power density. The photoresponse of 0.25, 0.32, 0.38, 0.41, 0.45, 0.52 A W^−1^ are achieved for the power tensities of 0.48, 0.56, 0.65, 0.71, 0.76, and 0.85 mW cm^−2^. In addition, the photodetectivity of bismuth oxyhydroxide and oxysulfide is 2.6 × 10^9^, 3.4 × 10^9^, 4.0 × 10^9^, 4.3 × 10^9^, 4.7 × 10^9^, and 5.0 × 10^9^ Jones for the power tensities of 0.48, 0.56, 0.65, 0.71, 0.76, and 0.85 mW cm^−2^. As shown in Figure 4e, the photoresponse and detectivity are fitted well with a linear curve for the power tensity. Figure 4f shows the time‐resolved photocurrent with periodical turning off‐on the UV light, it is found that the photocurrent presents little variation during fourteen off‐on photo‐switching cycles, indicating a good repeatability of Bi_2_O_0.4_S_2.6_‐based photodetector, which make it more favorable for practical application.

**Figure 5:**
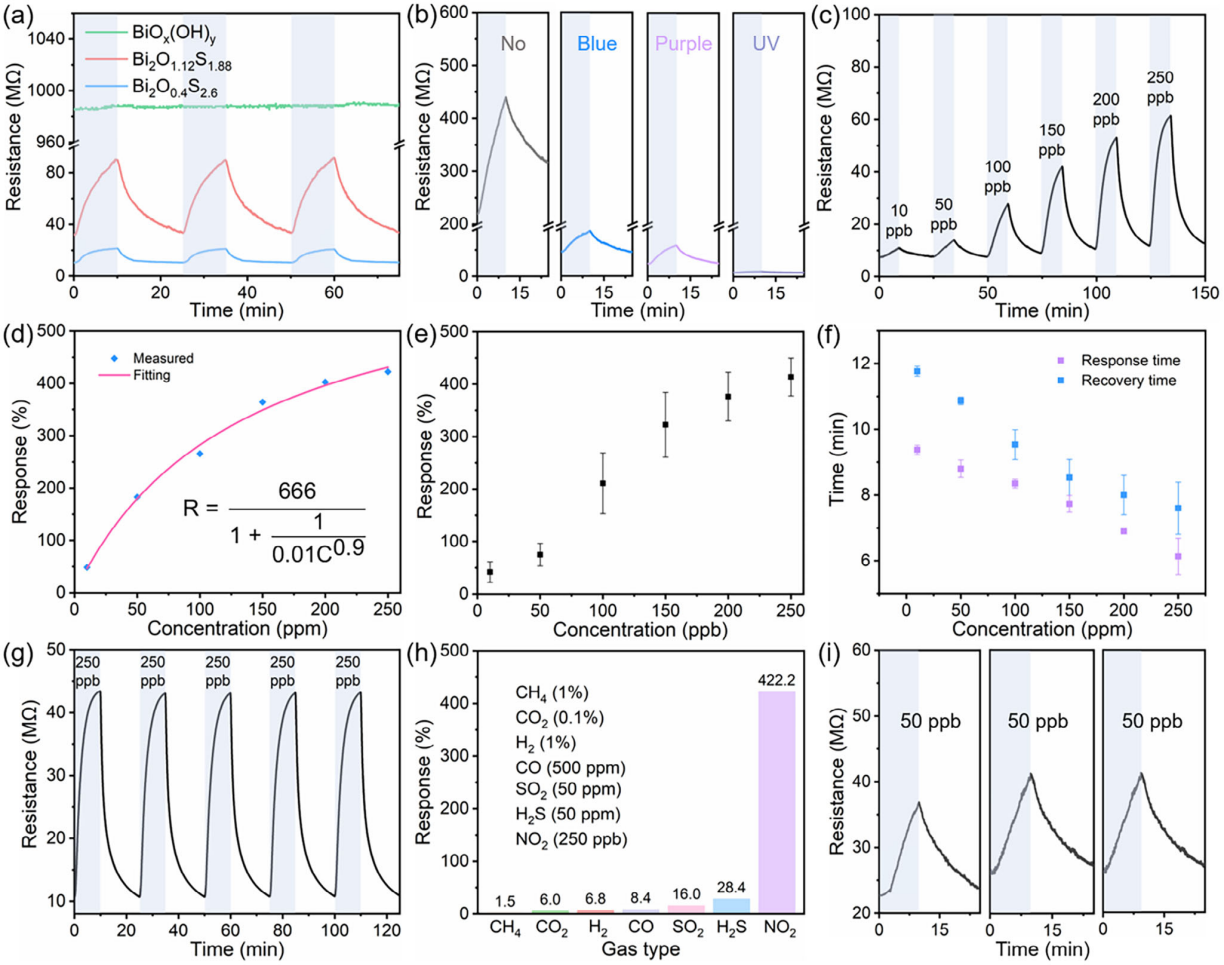
Performances of bismuth oxyhydroxide and oxysulfide‐based gas sensor. a) Resistance changes of bismuth oxyhydroxide and oxysulfide sensor with response to 250 ppb NO_2_ under purple light irradiation. b) Sensor response of Bi_2_O_1.22_S_1.88_ toward 100 ppb NO_2_ at various light irradiation. c) Dynamic gas response of Bi_2_O_1.22_S_1.88_ sensor toward different NO_2_ concentrations at purple light irradiation. d) Correlation between gas response and NO_2_ concentration for Bi_2_O_1.22_S_1.88_ sensor. e) Error analysis of the gas sensing response in **Figure** **5**c and Figure S14 (Supporting Information). f) Response and recovery time under different NO_2_ concentrations, the error bar is estimated by the statistic in Figure 5c and Figure S14 (Supporting Information). g) Repeatability of Bi_2_O_1.22_S_1.88_ sensor to 250 ppb NO_2_ under purple light activation at room temperature. h) Selectivity of Bi_2_O_1.22_S_1.88_ based gas sensor. i) Long‐term stability of Bi_2_O_1.22_S_1.88_ based gas sensor toward 50 ppb NO_2_.

Given the good optoelectronic properties of bismuth oxysulfide, the gas sensing performances toward NO_2_ under light irradiation have been investigated. The light with higher energy than the bandgap energy can excite more charge carriers to participate in interaction with gas molecules, thus replacing the heater activation to achieve room temperature gas sensing and improve the gas sensing response. Moreover, the photo energy can provide more reaction activation energy for the adsorption and desorption of gas molecules, thus improving the reversibility of the gas sensor.^[^ ^61^, ^62^ ^]^ A Great number of electron–hole pairs will be produced in bismuth oxysulfide after being exposed to light irradiation due to the higher energy of light compared with its bandgap energy ($hv\;\to \;h_{\left(hv\right)}^{-}+e_{\left(hv\right)}^{-}$). After that, NO_2_ molecules adsorb onto the surface of bismuth oxysulfide through physical interactions and draw the photogenerated electrons to form ${\mathrm{NO}}_{2}^{-}$ ($NO_{2}+e_{\left(hv\right)}^{-}\;\to \;NO_{2(hv)}^{-}$).^[^ ^63^, ^64^, ^65^ ^]^ The interfacial electron transfer is able to change the number of free charge carriers in bismuth oxysulfide, thus forming the gas‐sensing response. The gas response curves of the sensors based on bismuth oxyhydroxide and oxysulfide are depicted in Figure 5a. Under purple light irradiation, BiO_x_(OH) _y‐_based sensor does not exhibit any resistance change to 250 ppb NO_2_, probably because the wide energy bandgap leads to the absence of free electrons interacting with NO_2_ molecules. In contrast, the sensors based on bismuth oxysulfide present good gas response, in which the gas response of Bi_2_O_1.22_S_1.88_ and Bi_2_O_0.4_S_2.6_ to 250 ppb NO_2_ is 212.0% and 105.8%, respectively. Notably, the sensor based on Bi_2_O_1.22_S_1.88_ shows a better gas response than Bi_2_O_0.4_S_2.6_, while presenting a smaller photocurrent under the same light illumination. This phenomenon proves that the gas response is affected not only by the photoexcitation but also by the energy band structure, in which the photoexcitation generates more free electrons and the larger energy difference between the CBM of sensing materials and the LUMO of NO_2_ molecules supports stronger electrons transfer as shown in Figure S13 (Supporting Information).^[^ ^66^, ^67^ ^]^ Figure 5b displays the NO_2_ gas sensing resistance curves of Bi_2_O_1.22_S_1.88_ based sensor under different light irradiation environments. In a dark environment without any light irradiation, the base resistance is the highest among all sensors with a gas response of 39.2% and incomplete recovery. When light irradiation is introduced in the environment, the sensor exhibits decreased base resistance and full recovery due to the photoexcited carriers and photo‐desorption of NO_2_ molecules. The sensor presents the best gas response in purple light (149.1%) compared with blue (89.8%) and UV light (26.7%). The decreased gas response in UV light may be attributed to the excessive carrier density suppresses the exciton lifetime and more importantly high photo energy leads to the acceleration of photo‐desorption rate, thus resulting in the reduction of effective adsorption of NO_2_ molecules.^[^ ^68^, ^69^ ^]^ The degraded recovery time of the sensor as the excitation light changed from blue, purple, and UV light proves the acceleration of the photo‐desorption rate under high photon energy, as shown in Table S1 (Supporting Information).

The optoelectronic room temperature gas sensing performance of Bi_2_O_1.22_S_1.88_ based sensor has been further systematically investigated under purple light irradiation. As shown in Figure 5c, the dynamic gas responses of 48.5%, 182.9%, 265.8%, 364.1%, 402.3%, and 422.2% are achieved for the NO_2_ concentration of 10, 50, 100, 150, 200, and 250 ppb. The statistical analysis in Figure 5d indicates that the correlation between gas response and NO_2_ concentration fits well with a classic Langmuir Isotherm model. Moreover, the error analysis of the gas sensing response for Bi_2_O_1.22_S_1.88_ based sensor toward different NO_2_ concentrations is obtained through statistical analysis of the response in Figure 5c and Figure S14 (Supporting Information), in which the response of these sensors exhibits a good consistency as shown in Figure 5e. Compared with the reported bismuth compounds‐based NO_2_ gas sensor, the Bi_2_O_1.22_S_1.88_ sensor exhibits the lowest detection limit (**Table** **1**). The response and recovery time of the sensor toward various NO_2_ concentrations are represented in Figure 5f. The response and recovery time exhibit a decreasing trend with the increase of NO_2_ concentration, which benefits from the increased concentration reduced time for saturation adsorption on the surface of Bi_2_O_1.22_S_1.88_. Figure 5g reveals the repeatability of the sensor based on Bi_2_O_1.22_S_1.88_ to 250 ppb NO_2_ under purple light irradiation at room temperature. It is found that the baseline resistance of this gas sensor presents a negligible variation and the amplitude of dynamic resistance change remains the same during five continuous testing cycles, which indicates the good repeatability of this sensor. The selectivity of Bi_2_O_1.22_S_1.88_ based gas sensor toward seven common gases is shown in Figure 5h. The response to 250 ppb NO_2_ is significantly higher than the response to 50 ppm H_2_S, 50 ppm SO_2_, 500 ppm CO, 1% H_2_, 0.1% CO_2_, and 1% CH_4_. The high selectivity toward NO_2_ gas molecules is attributed to the stronger affinity of NO_2_ on the Bi_2_O_1.22_S_1.88_ surface, which leads to more NO_2_ molecules being able to adsorb on the surface. Moreover, there are more electrons transfer between Bi_2_O_1.22_S_1.88_ and NO_2_ molecules due to the favorable electronic band position.^[^ ^70^, ^71^ ^]^ The effect of ambient humidity on the gas sensing response to 250 ppb NO_2_ under purple light irradiation is investigated. As shown in Figure S15 (Supporting Information), the gas response decreased from 323.9% to 300.5%, and 235.4% as the ambient changed from dry condition to 24.6% RH, and 46.3% RH, which was possibly due to the competition adsorption between water and NO_2_ molecule leads to the reduction in the adsorption sites of NO_2_.^[^ ^72^, ^73^ ^]^ The long‐term stability of Bi_2_O_1.22_S_1.88_ based sensor has been studied by measuring the response to 50 ppb of NO_2_ as shown in Figure 5i. The sensor exhibits slightly increased base resistance from 25.3, 25.9 to 26.1 MΩ, while its response value maintains high consistency from 60.6%, 59.4% to 59.0% after one week and one month indicating good long‐term stability, which is beneficial for practical application.

**Table 1:** Summary of the detection limit for bismuth compounds based NO_2_ sensors.

Materials	Conc. [ppb]	Res.	Res. defined	O.T. [°C]	Refs.
Bi_2_O_3_	5000	5.8	*R_g_/R_0_*	RT	[74]
Bi_2_S_3_	500	3.2	*R_g_/R_0_*	RT	[75]
V_S_‐Bi_2_S_3_	25	1.25	*R_g_/R_0_*	RT	[76]
SnO_2_/Bi_2_O_3_	2000	56.92	*R_g_/R_0_*	250	[77]
ZnO/Bi_2_O_3_	500	3	*R_g_/R_0_*	300	[78]
Pt/Bi_2_O_3_	1000	141%	*(R_g_ – R_0_)/R_0_ × 100%*	300	[79]
Pt/SnO_2_/Bi_2_O_3_	1000	27.75	*R_g_/R_0_*	50	[80]
Au/Bi_2_S_3_	250	2	*R_g_/R_0_*	RT	[81]
ZnO/Bi_2_O_3_	1000	227%	*(R_g_ – R_0_)/R_0_ × 100%*	RT	[82]
MoS_2_/Bi_2_S_3_	30	1.05	*R_g_/R_0_*	RT	[83]
SnS_2_/Bi_2_S_3_	50	1.5	*R_g_/R_0_*	RT	[84]
BiOCl/Bi_2_S_3‐x_	20	1.3	*R_g_/R_0_*	RT	[85]
CuS/Bi_2_S_3_	500	1.4	*R_g_/R_0_*	RT	[86]
MoS_2_/Bi_2_O_3_/Bi_2_S_3_	50	1.1	*R_g_/R_0_*	RT	[87]
Bi_2_O_1.12_S_1.88_	10	48.5%	*(R_g_ – R_0_)/R_0_ × 100%*	RT	This work

Conc.: Detection limit concentration, Res.: Response, O.T.: Operating temperature, RT: Room temperature, Vs: sulfur vacancies.

## 3 Conclusion

The tunable synthesis of 2D bismuth oxyhydroxide and oxysulfide from bismuth metal was demonstrated in a solid–liquid reaction system. As sonicated in a pure aqueous environment, bismuth metal was able to react with dissolved oxygen and water molecules simultaneously, prompting an ultrathin self‐limiting oxyhydroxide layer to grow on its surface. Two types of bismuth oxysulfide were obtained in different concentrations of Na_2_S, which can introduce the dissolved H_2_S in aqueous environment to achieve the conversion from bismuth oxyhydroxide to oxysulfide. Benefiting from the lattice mismatch, the ultrasonic force enabled the surface layer to exfoliate from solid bismuth to form suspended 2D bismuth oxyhydroxide (BiO_x_(OH)_y_) and oxysulfide (Bi_2_O_1.12_S_1.88_, Bi_2_O_0.4_S_2.6_) nanosheets. The morphology characterization reveals that both bismuth oxyhydroxide and oxysulfide exhibit 2D morphology. The introduction of S atoms in bismuth oxysulfide achieved the modulation of electronic structures, resulting in a redshift optical absorption from UV (≈370 nm) to blue light (≈550 nm) region compared with oxyhydroxide. The photodetectors based on bismuth oxysulfide exhibit the broad response from blue to purple and UV light. Given the good photoresponse, the performance of bismuth oxysulfide‐based gas sensor has been investigated at room temperature under visible light irradiation. The sensor based on Bi_2_O_1.12_S_1.88_ exhibited a higher response (212.0%) compared with Bi_2_O_0.4_S_2.6_ (105.8%) to 250 ppb NO_2_, attributing that the largest energy difference between the CBM of Bi_2_O_1.12_S_1.88_ and the LOMO of NO_2_ molecules leads to more electrons transfer. Moreover, the Bi_2_O_1.12_S_1.88_‐based sensor presented a reversible gas response toward ultra‐low NO_2_ concentration from 10, 50, 100, 150, 200 to 250 ppb under purple light irradiation. While, the sensor has also been proven to have excellent repeatability, selectivity, and long‐term stability. This research achieves the effective synthesis of 2D bismuth oxyhydroxide and oxysulfide that can be extended to other metal oxyhydroxide and oxysulfide and realizes the application of bismuth oxysulfide in photodetection and gas sensing.

## 4 Experimental Section

### The Synthesis of 2D Bismuth Oxyhydroxide and Oxysulfide

The bismuth oxyhydroxide was synthesized via sonicating solid bismuth in a pure 25% ethanol solution. First, 5 g bulk solid bismuth particles (Aladdin, 99.99%) with an average diameter of 2 mm were added into 40 mL 25%v/v ethanol. Then, the bismuth particle suspension was kept bath sonicated (KQ‐300DE) for 2 h at 86 °C. After that, the dispersion was centrifuged for 30 min at 4000 RPM (GL‐20B). Next, the supernatant was centrifuged for 30 min at 12 000 RPM and redispersed in 25%v/v ethanol. The bismuth oxysulfide was synthesized by sonicating bismuth particles in Na_2_S solutions with different concentrations (i.e., 0.1, 1 mol L^−1^). The different concentrations of Na_2_S solution were obtained via dissolving Na_2_S 9H_2_O (Aladdin, 99.99%) in 25%v/v ethanol solvent. Then, bismuth particles in Na_2_S solution were also kept bath sonicated for 2 h at 86 °C. Finally, the dispersion after sonicated was centrifuged as above parameters.

### Characterizations

Scanning electron microscopy (SEM, JEM‐2100F), atomic force microscopy (AFM, Park NX 10), and transmission electron microscopy (TEM, JEOL‐2100F) were used to characterize the morphology of bismuth oxyhydroxide and oxysulfide. The crystal structure was revealed by high‐resolution transmission electron microscopy (HRTEM, JEOL‐2100F) and selected area electron diffraction (SAED). The elemental and chemical states were characterized through energy‐dispersive X‐ray spectroscopy (EDS), and X‐ray photoelectron spectrometer (XPS, Thermo Scientific K‐Alpha Plus). Raman spectra were collected by HORIBA LabRAM HR Evolution with a laser source of 532 nm. Fourier‐transform infrared (FTIR) spectra were recorded on a Nicolet iS50 infrared spectrometer in the range of 400–4000 cm^−1^ using pressed KBr discs. The energy band structure was obtained via the spectrophotometer (Shimadzu UV‐2600) and X‐ray photoelectron spectrometer (Thermo Scientific K‐Alpha Plus). Mott–Schottky measurements were conducted using the CHI660E electrochemical workstation.

### Device Fabrication and Measurements

The transducing substrates consisted of 10 µm wide circular interdigitated electrodes (IDEs) were made by a standard micro‐nano manufacturing technology. Photodetectors and gas sensors were obtained through drop‐casting 40 µL dispersion containing 2D bismuth oxyhydroxide and oxysulfide on the IDEs at 45 °C. The photoresponse measurements were conducted under UV (365 nm), purple (405 nm), and blue (450 nm) light illumination. The photocurrent was collected using a Tektronix 2602B digital source meter with 5 V bias voltages. The sensor resistance was obtained using a Tektronix DMM 4040 digital multimeter. The total gas flow rate of the testing chamber was maintained at 200 sccm through the Alicat MC‐200 mass flow controller. The gas response is defined as *(R_g_ – R_0_) / R_0_ × 100%* for the oxidizing gas and (*R*_0_  −  *R_g_*) / *R_g_*  ×  100% for the reducing gas, where *R_g_* and *R*_0_ is the resistance of the sensor in analyte gas and balance gas environment. The time for reaching 90% of the highest resistance variation after exposure to NO_2_ and N_2_ gas were defined as the response and recovery time.

## Conflict of Interest

The authors declare no conflict of interest.

## Supporting information



Supporting Information

## Data Availability

The data that support the findings of this study are available from the corresponding author upon reasonable request.

## References

[1] N. Cabrera , N. F. Mott , Rep. Prog. Phys. 1948, 12, 163.

[2] H. Over , A. P. Seitsonen , Science 2002, 297, 2003. 12242427 10.1126/science.1077063

[3] P. Aukarasereenont , A. Goff , C. K. Nguyen , C. F. McConville , A. Elbourne , A. Zavabeti , T. Daeneke , Chem. Soc. Rev. 2022, 51, 1253. 35107468 10.1039/d1cs01166a

[4] S. Zhao , J. Zhang , L. Fu , Adv. Mater. 2021, 33, 2005544. 10.1002/adma.202005544 33448060

[5] A. Goff , P. Aukarasereenont , C. K. Nguyen , R. Grant , N. Syed , A. Zavabeti , A. Elbourne , T. Daeneke , Dalton Trans. 2021, 50, 7513. 33977926 10.1039/d0dt04364h

[6] T. Daeneke , P. Atkin , R. Orrell‐Trigg , A. Zavabeti , T. Ahmed , S. Walia , M. Liu , Y. Tachibana , M. Javaid , A. D. Greentree , S. P. Russo , R. B. Kaner , K. Kalantar‐Zadeh , ACS Nano 2017, 11, 10974. 29045121 10.1021/acsnano.7b04856

[7] A. Zavabeti , J. Z. Ou , B. J. Carey , N. Syed , R. Orrell‐Trigg , E. L. H. Mayes , C. L. Xu , O. Kavehei , A. P. O'Mullane , R. B. Kaner , K. Kalantar‐Zadeh , T. Daeneke , Science 2017, 358, 332. 29051372 10.1126/science.aao4249

[8] K. A. Messalea , B. J. Carey , A. Jannat , N. Syed , M. Mohiuddin , B. Y. Zhang , A. Zavabeti , T. Ahmed , N. Mahmood , E. Della Gaspera , K. Khoshmanesh , K. Kalantar‐Zadeh , T. Daeneke , Nanoscale 2018, 10, 15615. 30090912 10.1039/c8nr03788d

[9] A. Zavabeti , P. Aukarasereenont , H. Tuohey , N. Syed , A. Jannat , A. Elbourne , K. A. Messalea , B. Y. Zhang , B. J. Murdoch , J. G. Partridge , M. Wurdack , D. L. Creedon , J. van Embden , K. Kalantar‐Zadeh , S. P. Russo , C. F. McConville , T. Daeneke , Nat. Electron. 2021, 4, 277.

[10] Y. Cheng , Z. Li , L. Cheng , Y. Yuan , E. Xie , X. Cao , Z. Xin , Y. Liu , T. Tang , X. Hu , K. Xu , C. M. Hung , A. Jannat , Y. X. Li , H. Chen , J. Z. Ou , ACS Appl. Mater. Interfaces 2023, 15, 57496. 10.1021/acsami.3c12787 38015181

[11] K. A. Messalea , N. Syed , A. Zavabeti , M. Mohiuddin , A. Jannat , P. Aukarasereenont , C. K. Nguyen , M. X. Low , S. Walia , B. Haas , C. T. Koch , N. Mahmood , K. Khoshmanesh , K. Kalantar‐Zadeh , T. Daeneke , ACS Nano 2021, 15, 16067. 34623147 10.1021/acsnano.1c04631

[12] B. Y. Zhang , K. Xu , Q. Yao , A. Jannat , G. Ren , M. R. Field , X. Wen , C. Zhou , A. Zavabeti , J. Z. Ou , Nat. Mater. 2021, 20, 1073. 33462466 10.1038/s41563-020-00899-9

[13] J. N. Coleman , M. Lotya , A. O'Neill , S. D. Bergin , P. J. King , U. Khan , K. Young , A. Gaucher , S. De , R. J. Smith , I. V. Shvets , S. K. Arora , G. Stanton , H. Y. Kim , K. Lee , G. T. Kim , G. S. Duesberg , T. Hallam , J. J. Boland , J. J. Wang , J. F. Donegan , J. C. Grunlan , G. Moriarty , A. Shmeliov , R. J. Nicholls , J. M. Perkins , E. M. Grieveson , K. Theuwissen , D. W. McComb , P. D. Nellist , et al., Science 2011, 331, 568. 21292974 10.1126/science.1194975

[14] V. Nicolosi , M. Chhowalla , M. G. Kanatzidis , M. S. Strano , J. N. Coleman , Science 2013, 340, 1226419.

[15] X. Zhang , J. Liu , Z. Deng , Mater. Horiz. 2024, 11, 1369. 38224183 10.1039/d3mh01722b

[16] S. Zhang , S. Guo , Z. Chen , Y. Wang , H. Gao , J. Gómez‐Herrero , P. Ares , F. Zamora , Z. Zhu , H. Zeng , Chem. Soc. Rev. 2018, 47, 982. 29210397 10.1039/c7cs00125h

[17] H. Lu , Q. Hao , T. Chen , L. Zhang , D. Chen , C. Ma , W. Yao , Y. Zhu , Appl. Catal. B: Environ. 2018, 237, 59.

[18] Y. Wang , W. Feng , M. Chang , J. Yang , Y. Guo , L. Ding , L. Yu , H. Huang , Y. Chen , J. Shi , Adv. Funct. Mater. 2020, 31, 2005093.

[19] A. S. Mogoda , K. M. Zohdy , Int. J. Electrochem. Sci. 2022, 17, 22012.

[20] O. N. Fedyaeva , A. P. Grebennikov , A. A. Vostrikov , J. Eng. Thermophys. 2024, 33, 467.

[21] C. K. Nguyen , M. X. Low , A. Zavabeti , A. Jannat , B. J. Murdoch , E. Della Gaspera , R. Orrell‐Trigg , S. Walia , A. Elbourne , V. K. Truong , C. F. McConville , N. Syed , T. Daeneke , J. Mater. Chem. C 2021, 9, 11815.

[22] Y. Zhang , H. Wu , Z. Hu , H. Yu , Chem. Eur. J. 2023, 29, e202203597. 36524850 10.1002/chem.202203597

[23] Q. Wang , M. Nakabayashi , T. Hisatomi , S. Sun , S. Akiyama , Z. Wang , Z. Pan , X. Xiao , T. Watanabe , T. Yamada , N. Shibata , T. Takata , K. Domen , Nat. Mater. 2019, 18, 827. 31209390 10.1038/s41563-019-0399-z

[24] S. Tippireddy , P. K. D. S. , S. Das , R. C. Mallik , ACS Appl. Energ. Mater. 2021, 4, 2022.

[25] E. A. Bondarenko , E. A. Streltsov , M. V. Malashchonak , A. V. Mazanik , A. I. Kulak , E. V. Skorb , Adv. Mater. 2017, 29, 1702387. 10.1002/adma.201702387 28850736

[26] F. Wang , S. Yang , J. Wu , X. Hu , Y. Li , H. Li , X. Liu , J. Luo , T. Zhai , InfoMat. 2021, 3, 1251.

[27] D. A. Giannakoudakis , M. Jiang , T. J. Bandosz , ACS Appl. Mater. Interfaces 2016, 8, 31986. 27800680 10.1021/acsami.6b10544

[28] T. Raabe , M. Mehne , H. Rasser , H. Krause , S. Kureti , Chem. Eng. J. 2019, 371, 738.

[29] S. Lee , M. Govindan , D. Kim , Chem. Eng. J. 2021, 416, 127918.

[30] Y. Hernandez , V. Nicolosi , M. Lotya , F. M. Blighe , Z. Sun , S. De , I. T. McGovern , B. Holland , M. Byrne , Y. K. Gun'Ko , J. J. Boland , P. Niraj , G. Duesberg , S. Krishnamurthy , R. Goodhue , J. Hutchison , V. Scardaci , A. C. Ferrari , J. N. Coleman , Nat. Nanotechnol. 2008, 3, 563. 18772919 10.1038/nnano.2008.215

[31] T. W. Kim , K. H. Park , Y. E. Choi , J. Y. Lee , Y. S. Jung , J. Mater. Chem. A 2018, 6, 840.

[32] K. Li , Y. Cai , X. Yang , S. Wang , C. Teng , Y. Tian , Q. Min , W. Zhu , Adv. Funct. Mater. 2022, 32, 2113002.

[33] S. K. Singh , A. Kumar , B. Gahtori , Shruti , G. Sharma , S. Patnaik , V. P. S. Awana , J. Am. Chem. Soc. 2012, 134, 16504. 23003214 10.1021/ja307245a

[34] J. Liu , Y. Yang , B. Ni , H. Li , X. Wang , Small 2016, 13, 1602637.

[35] H. Huang , Y. He , X. Li , M. Li , C. Zeng , F. Dong , X. Du , T. Zhang , Y. Zhang , J. Mater. Chem. A 2015, 3, 24547.

[36] L. Hao , L. Kang , H. Huang , L. Ye , K. Han , S. Yang , H. Yu , M. Batmunkh , Y. Zhang , T. Ma , Adv. Mater. 2019, 31, 1900546. 10.1002/adma.201900546 31058378

[37] T. Han , X. Cao , K. Sun , Q. Peng , C. Ye , A. Huang , W.‐C. Cheong , Z. Chen , R. Lin , D. Zhao , X. Tan , Z. Zhuang , C. Chen , D. Wang , Y. Li , Nat. Commun. 2021, 12, 4952. 34400649 10.1038/s41467-021-25261-8 PMC8368037

[38] Y. Yang , Z. Bian , L. Zhang , H. Wang , J. Hazard. Mater. 2022, 427, 127866. 34857401 10.1016/j.jhazmat.2021.127866

[39] X. Wang , M. Xie , F. Lyu , Y.‐M. Yiu , Z. Wang , J. Chen , L.‐Y. Chang , Y. Xia , Q. Zhong , M. Chu , H. Yang , T. Cheng , T.‐K. Sham , Q. Zhang , Nano Lett. 2020, 20, 7751. 32959660 10.1021/acs.nanolett.0c03340

[40] D. Liu , J. Zhang , C. Li , X. Zhang , X. Chen , F. Wang , M. Shi , R. Li , C. Li , Appl. Catal. B: Environ. 2019, 248, 459.

[41] Y. Lu , Y. Huang , Y. Zhang , J.‐j. Cao , H. Li , C. Bian , S. C. Lee , Appl. Catal. B: Environ. 2018, 231, 357.

[42] M. Weber , R. D. Rodriguez , D. R. T. Zahn , M. Mehring , Inorg. Chem. 2018, 57, 8540. 29949355 10.1021/acs.inorgchem.8b01249

[43] H. Liu , M. Luo , J. Hu , T. Zhou , R. Chen , J. Li , Appl. Catal. B: Environ. 2013, 140–141, 141.

[44] I. A. T. Gaia , E. V. Guimarães , P. I. S. Maia , H. D. Mikhail , M. S. da Luz , A. C. A. S. , R. S. Silva , Physica B 2023, 662, 414947.

[45] M. A. Hobosyan , S. A. Yolchinyan , K. S. Martirosyan , RSC Adv. 2016, 6, 66564.

[46] Y. Sun , W. Wang , L. Zhang , Z. Zhang , Chem. Eng. J. 2012, 211–212, 161.

[47] G. S. Bang , K. W. Nam , J. Y. Kim , J. Shin , J. W. Choi , S.‐Y. Choi , ACS Appl. Mater. Interfaces 2014, 6, 7084. 24773226 10.1021/am4060222

[48] Y. Z. Yang , H. Liang , X. Wang , X. Ma , T. Zhang , Y. Yang , L. Xie , D. Chen , Y. Long , J. Chen , Y. Chang , C. Yan , X. Zhang , X. Zhang , B. Ge , Z. Ren , M. Xue , G. Chen , ACS Nano 2015, 10, 755. 26690902 10.1021/acsnano.5b05823

[49] Q. Gong , L. Cheng , C. Liu , M. Zhang , Q. Feng , H. Ye , M. Zeng , L. Xie , Z. Liu , Y. Li , ACS Catal. 2015, 5, 2213.

[50] S. R. Kadam , R. P. Panmand , R. S. Sonawane , S. W. Gosavi , B. B. Kale , RSC Adv. 2015, 5, 58485.

[51] J. Ke , J. Liu , H. Sun , H. Zhang , X. Duan , P. Liang , X. Li , M. O. Tade , S. Liu , S. Wang , Appl. Catal. B: Environ. 2017, 200, 47.

[52] B. Shao , X. Liu , Z. Liu , G. Zeng , Q. Liang , C. Liang , Y. Cheng , W. Zhang , Y. Liu , S. Gong , Chem. Eng. J. 2019, 368, 730.

[53] Y. Sang , X. Cao , G. Dai , L. Wang , Y. Peng , B. Geng , J. Hazard. Mater. 2020, 381, 120942. 31416040 10.1016/j.jhazmat.2019.120942

[54] M. B. Ghasemian , A. Zavabeti , M. Mousavi , B. J. Murdoch , A. J. Christofferson , N. Meftahi , J. Tang , J. Han , R. Jalili , F. M. Allioux , M. Mayyas , Z. Chen , A. Elbourne , C. F. McConville , S. P. Russo , S. Ringer , K. Kalantar‐Zadeh , Adv. Mater. 2021, 33, 2104793. 10.1002/adma.202104793 34510605

[55] T. Tang , Z. Li , Y. Y. Liu , Y. L. Chen , Y. F. Cheng , Y. Liang , J. H. Zhuang , X. Y. Hu , A. Jannat , R. Ou , K. Xu , J. Z. Ou , Ceram. Int. 2025, 51, 3216.

[56] H.‐R. Fuh , C.‐R. Chang , Y.‐K. Wang , R. F. L. Evans , R. W. Chantrell , H.‐T. Jeng , Sci. Rep. 2016, 6, 32625. 27601195 10.1038/srep32625 PMC5013522

[57] A. Ishikawa , T. Takata , J. N. Kondo , M. Hara , H. Kobayashi , K. Domen , J. Am. Chem. Soc. 2002, 124, 13547. 12418910 10.1021/ja0269643

[58] A. Ishikawa , T. Takata , T. Matsumura , J. N. Kondo , M. Hara , H. Kobayashi , K. Domen , J. Phys. Chem. B 2004, 108, 2637.

[59] M. M. Y. A. Alsaif , S. Kuriakose , S. Walia , N. Syed , A. Jannat , B. Y. Zhang , F. Haque , M. Mohiuddin , T. Alkathiri , N. Pillai , T. Daeneke , J. Z. Ou , A. Zavabeti , Adv. Mater. Interfaces 2019, 6, 1900007.

[60] G. Y. Chen , X. Y. Hu , M. W. Gu , H. Wu , K. Y. Chen , H. Yu , B. Y. Ren , Z. Li , Y. G. Luan , T. Tang , Y. F. Cheng , H. B. Huang , L. G. Chen , B. Y. Zhang , J. Z. Ou , Adv. Funct. Mater. 2022, 32, 2202239.

[61] D. Gu , X. Y. Wang , W. Liu , X. G. Li , S. W. Lin , J. Wang , M. N. Rumyantseva , A. M. Gaskov , S. A. Akbar , Sens. Actuator B‐Chem. 2020, 305, 9.

[62] R. Kumar , N. Goel , M. Kumar , ACS Sens. 2017, 2, 1744. 29090571 10.1021/acssensors.7b00731

[63] J. Z. Ou , W. Ge , B. Carey , T. Daeneke , A. Rotbart , W. Shan , Y. Wang , Z. Fu , A. F. Chrimes , W. Wlodarski , S. P. Russo , Y. X. Li , K. Kalantar‐Zadeh , ACS Nano 2015, 9, 10313. 26447741 10.1021/acsnano.5b04343

[64] T. Tang , Z. Li , Y. F. Cheng , H. G. Xie , X. X. Wang , Y. L. Chen , L. Cheng , Y. Liang , X. Y. Hu , C. M. Hung , N. D. Hoa , H. Yu , B. Y. Zhang , K. Xu , J. Z. Ou , J. Hazard. Mater. 2023, 451, 131184. 36933506 10.1016/j.jhazmat.2023.131184

[65] M. Zhang , Z. Li , T. Tang , R. Ou , B. Y. Zhang , Q. Ma , Y. F. Cheng , Y. Liang , J. H. Zhuang , W. J. Zhang , A. Jannat , A. A. Haidry , J. Z. Ou , J. Alloy. Compd. 2025, 1010, 177562.

[66] T. Wang , B. Jiang , Q. Yu , X. Kou , P. Sun , F. Liu , H. Lu , X. Yan , G. Lu , ACS Appl. Mater. Interfaces 2019, 11, 9600. 30724073 10.1021/acsami.8b21543

[67] H. Chen , Y. Zhao , L. Shi , G. D. Li , L. Sun , X. Zou , ACS Appl. Mater. Interfaces 2018, 10, 29795. 30095885 10.1021/acsami.8b10057

[68] Y. F. Cheng , Z. Li , M. Zhang , H. G. Xie , T. Tang , Y. Liang , X. X. Wang , K. Xu , B. Y. Zhang , A. A. Haidry , J. Z. Ou , J. Mater. Chem. C 2023, 11, 14187.

[69] R. J. Chen , N. R. Franklin , J. Kong , J. Cao , T. W. Tombler , Y. Zhang , H. Dai , Appl. Phys. Lett. 2001, 79, 2258.

[70] T. Tang , Z. Li , Y. F. Cheng , K. Xu , H. G. Xie , X. X. Wang , X. Y. Hu , H. Yu , B. Y. Zhang , X. Tao , C. M. Hung , N. D. Hoa , G. Y. Chen , Y. Li , J. Z. Ou , J. Mater. Chem. A 2023, 11, 6361.

[71] K. Xu , B. Y. Zhang , M. Mohiuddin , N. Ha , X. M. Wen , C. H. Zhou , Y. X. Li , G. H. Ren , H. J. Zhang , A. Zavabeti , J. Z. Ou , Nano Today 2021, 37, 101096.

[72] R. Kumar , O. Al‐Dossary , G. Kumar , A. Umar , Nano‐Micro Lett. 2015, 7, 97. 10.1007/s40820-014-0023-3 PMC6223925 30464961

[73] T. Alkathiri , K. Xu , B. Y. Zhang , M. W. Khan , A. Jannat , N. Syed , A. F. M. Almutairi , N. Ha , M. M. Y. A. Alsaif , N. Pillai , Z. Li , T. Daeneke , J. Z. Ou , Small Sci. 2021, 2, 2100097. 40213279 10.1002/smsc.202100097 PMC11935894

[74] X. Gou , R. Li , G. Wang , Z. Chen , D. Wexler , Nanotechology 2009, 20, 495501. 10.1088/0957-4484/20/49/495501 19893148

[75] H. Kan , M. Li , Z. Song , S. Liu , B. Zhang , J. Liu , M.‐Y. Li , G. Zhang , S. Jiang , H. Liu , J. Colloid Interface. Sci. 2017, 506, 102. 28728027 10.1016/j.jcis.2017.07.012

[76] Y. Yang , T. Xin , C. Liu , T. Zhang , W. Hao , Y. Wang , J. Hao , J. Alloy. Compd. 2023, 930, 167467.

[77] J. H. Bang , M. S. Choi , A. Mirzaei , Y. J. Kwon , S. S. Kim , T. W. Kim , H. W. Kim , Sens. Actuators B Chem 2018, 274, 356.

[78] V. Ramakrishnan , K. G. Nair , J. Dhakshinamoorthy , K. R. Ravi , B. Pullithadathil , Phys. Chem. Chem. Phys. 2020, 22, 7524. 32219238 10.1039/d0cp00567c

[79] S. Park , S. An , H. Ko , C. Lee , J. Nanosci. Nanotechnol. 2015, 15, 1605. 26353699 10.1166/jnn.2015.9304

[80] J. H. Bang , A. Mirzaei , S. Han , H. Y. Lee , K. Y. Shin , S. S. Kim , H. W. Kim , Ceram. Int. 2021, 47, 5099.

[81] X. Chen , J. Shi , T. Wang , S. Zheng , W. Lv , X. Chen , J. Yang , M. Zeng , N. Hu , Y. Su , H. Wei , Z. Zhou , Z. Yang , ACS Sens. 2022, 7, 816. 35188381 10.1021/acssensors.1c02452

[82] S. Park , H. Ko , S. Lee , H. Kim , C. Lee , Thin Solid Films 2014, 570, 298.

[83] L. Liu , Y. Gao , Q. Hu , Appl. Nano Mater. 2023, 6, 8260.

[84] T. Wang , J. Liu , Y. Zhang , Q. Liang , R. Wu , H.‐S. Tsai , Y. Wang , J. Hao , J. Mater. Chem. A 2022, 10, 4306.

[85] Y. Yang , J. Mao , D. Yin , T. Zhang , C. Liu , W. Hao , Y. Wang , J. Hao , J. Hazard. Mater. 2023, 455, 131591. 37172379 10.1016/j.jhazmat.2023.131591

[86] X. Chen , T. Wang , J. Shi , W. Lv , Y. Han , M. Zeng , J. Yang , N. Hu , Y. Su , H. Wei , Z. Zhou , Z. Yang , Y. Zhang , Nano‐Micro Lett. 2021, 14, 8. 10.1007/s40820-021-00740-1 PMC8639894 34859321

[87] M. Ikram , L. Liu , H. Lv , Y. Liu , A. Ur Rehman , K. Kan , W. Zhang , L. He , Y. Wang , R. Wang , K. Shi , J. Hazard. Mater. 2019, 363, 335. 30321838 10.1016/j.jhazmat.2018.09.077

