# Supplementary material for: Tunable Synthesis of 2D Bismuth Oxyhydroxide and Oxysulfide from Solid–Liquid Interfacial Reaction for High Performance Optoelectronic Gas Sensing
Source: Small. 2025 Mar 10;21(17):2411522. doi: 10.1002/smll.202411522 (PMC12036554; doi:10.1002/smll.202411522)
Supplement: Supplementary file 1 — Supporting Information [file SMLL-21-2411522-s001.docx]

Supplementary materials for

**Tunable synthesis of two-dimensional bismuth oxyhydroxide and oxysulfide from solid-liquid interfacial reaction for high performance optoelectronic gas sensing**

Tao Tang^1^, Zhong Li^1,2^ *, Li Zhou^3^, Pu Zhang^3^, Yin Fen Cheng^4^, Yi Liang^1^, Jing Hao Zhuang^1^, Xin Yi Hu^1^, Qi Jie Ma^5^, Bao Yue Zhang^5^, Azmira Jannat^1,5^, and Jian Zhen Ou^1, 5^ *

^1^Key Laboratory of Advanced Technologies of Materials, Ministry of Education, School of Materials Science and Engineering, Southwest Jiaotong University, Chengdu 610031, China

^2^Jiangsu Key Laboratory of Advanced Structural Materials and Application Technology, Nanjing Institute of Technology, Nanjing 211167, China

^3^Research Institute of Natural Gas Technology, PetroChina Southwest Oil and Gas field Company, Chengdu 610213, China

^4^Institute of Advanced Study, Chengdu University, Chengdu 610106, China

^5^School of Engineering, RMIT University, Melbourne, Victoria 3000, Australia

*Corresponding author. E-mail: zhong.li@swjtu.edu.cn (Z. Li), jzou@swjtu.edu.cn, jianzhen.ou@rmit.edu.au (J. Z. Ou)


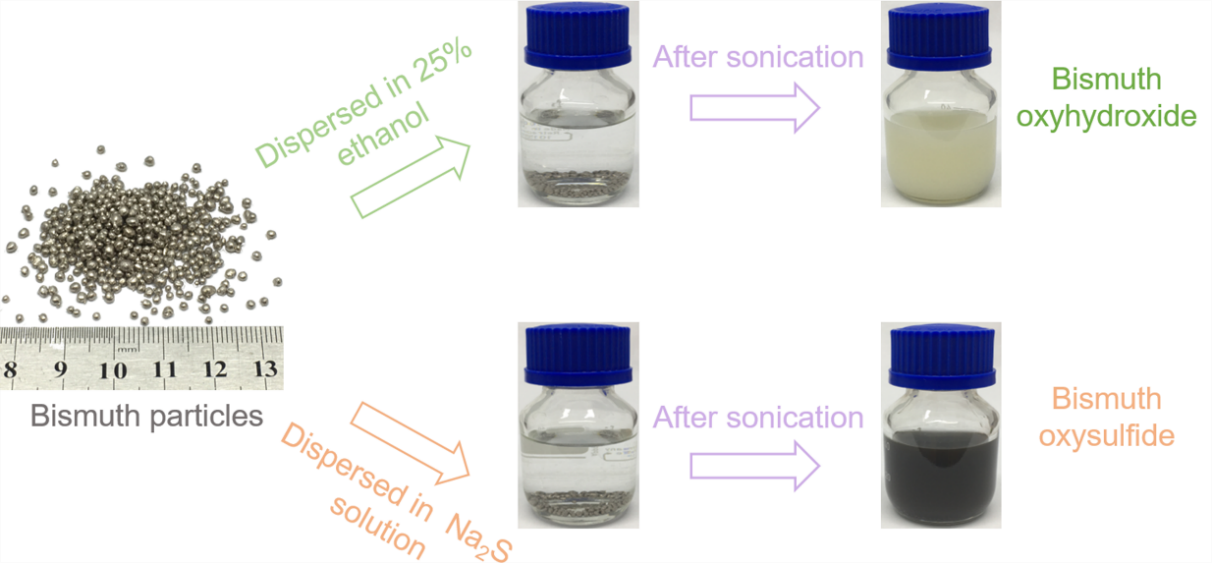


**Figure S1.** The photographs of bulk solid bismuth particles, ultrasonic aqueous environment and obtained bismuth oxyhydroxide and oxysulfide.


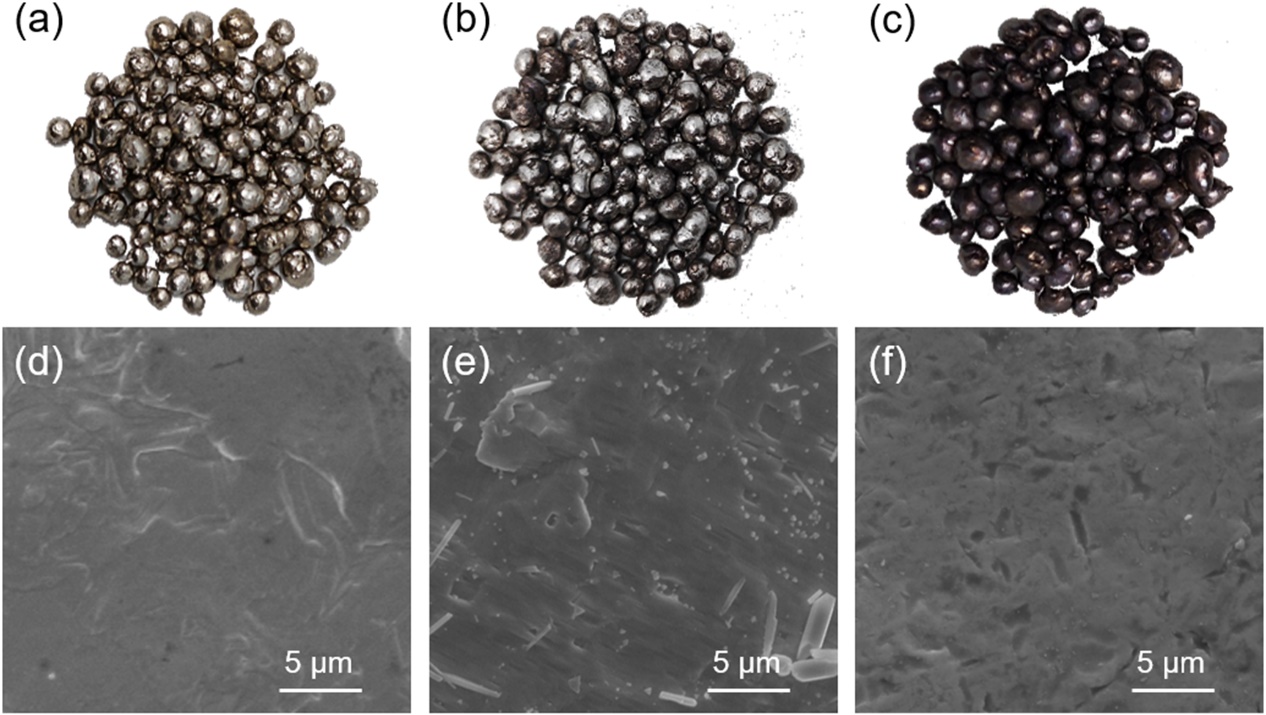


**Figure S2.** a) and d) the photograph and SEM imagine of the particle purchased from Aladdin without further processing. b) and e) the photograph and SEM imagine of the particle after sonicated in 25%v/v ethanol solution. c) and f) the photograph and SEM imagine of the particle after sonicated in 0.1 mol/L Na_2_S solution.


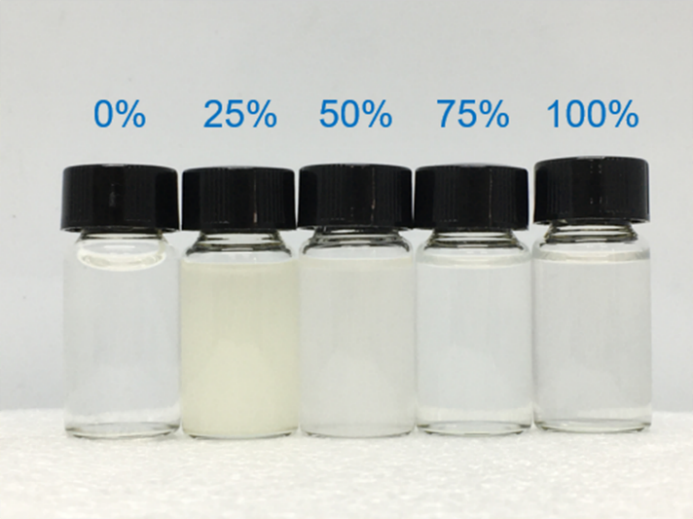


**Figure S3.** The photograph of bismuth oxyhydroxide suspension obtained in the mixed solution containing 0, 25, 50, 75, 100 %v/v ethanol.


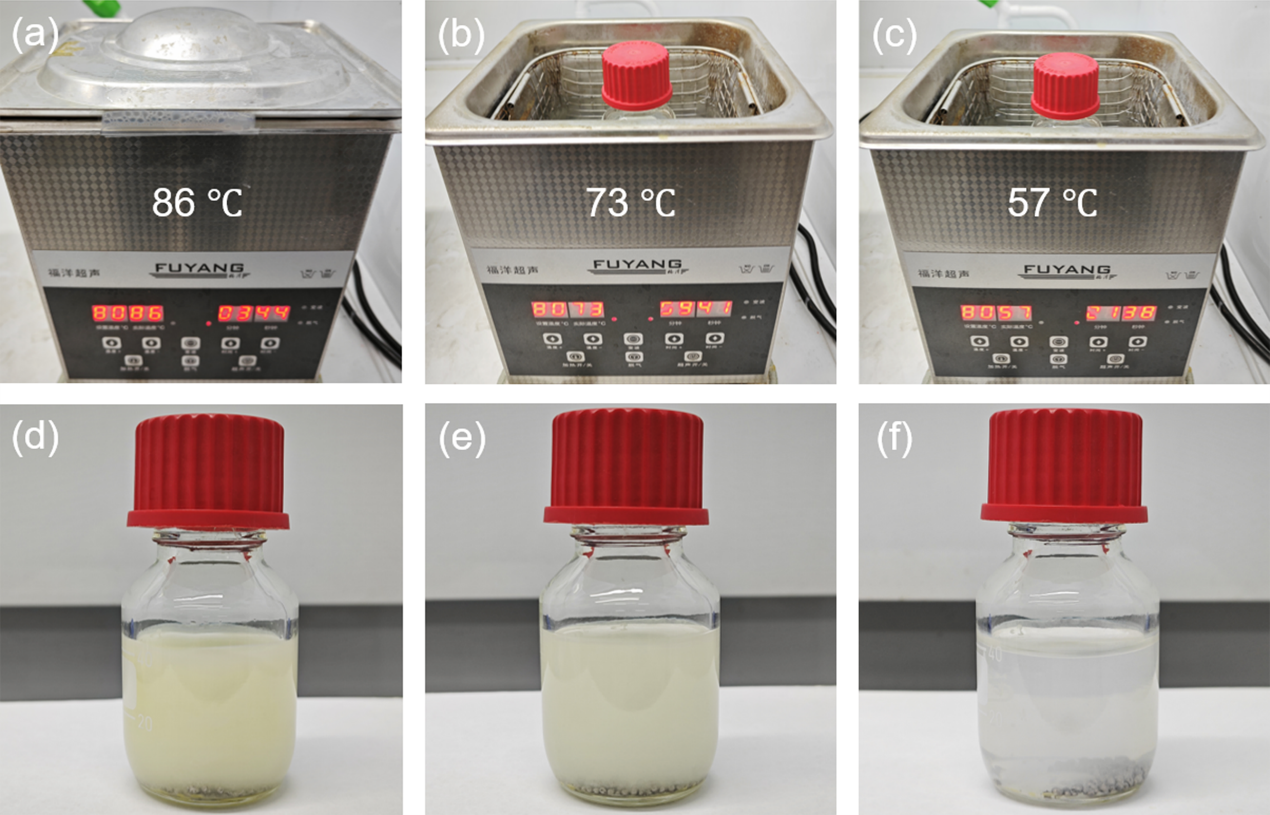


**Figure S4.** a)-c) The ultrasonic temperature control at 86 ℃, 73 ℃, and 57 ℃ is achieved by closing the cover of the ultrasound machine and turning on heating at the same time, opening the cover of the ultrasound machine and turning on heating, and just opening the lid of the ultrasound machine, respectively. d)-f) The dispersion of bismuth oxyhydroxide obtained from sonicated bismuth particles at 86 ℃, 73 ℃, and 57 ℃, respectively.


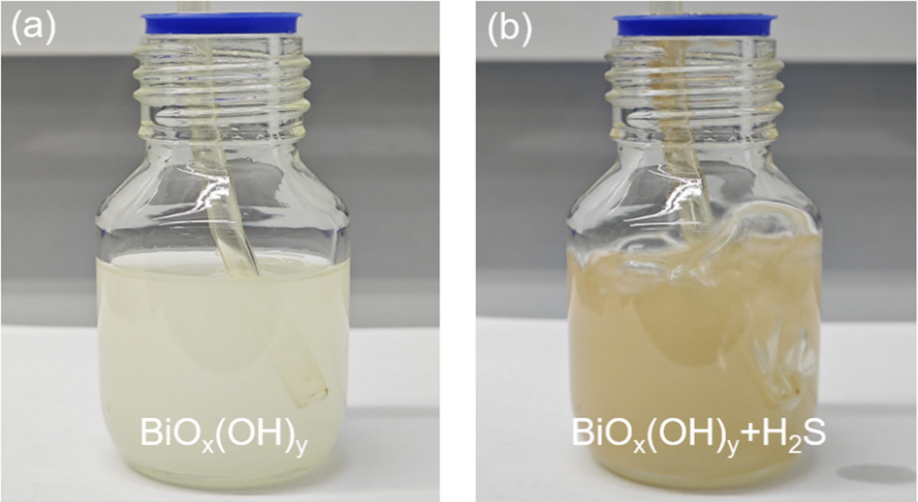


**Figure S5.** a) The dispersion of bismuth oxyhydroxide obtained from sonicated bismuth particles in 25%v/v ethanol. b) The change of bismuth hydroxide dispersion after injected 500 ppm H_2_S gas.


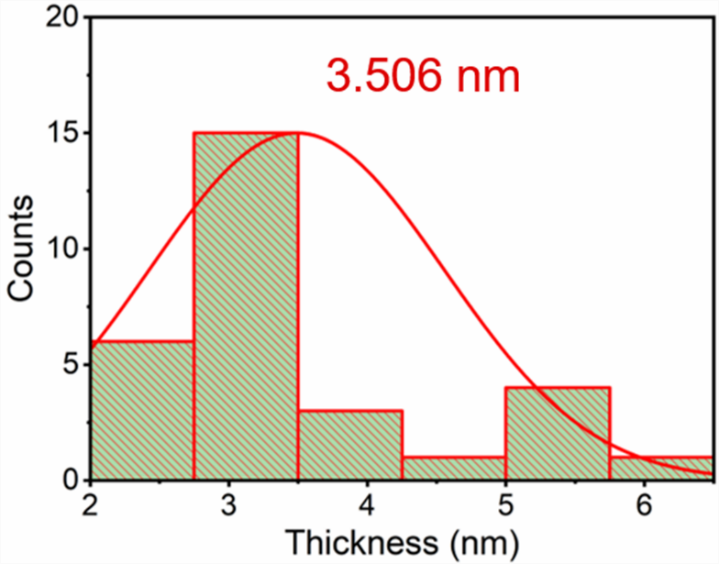


**Figure S6.** The statistical distribution of the thickness of 2D bismuth oxyhydroxide nanosheets.


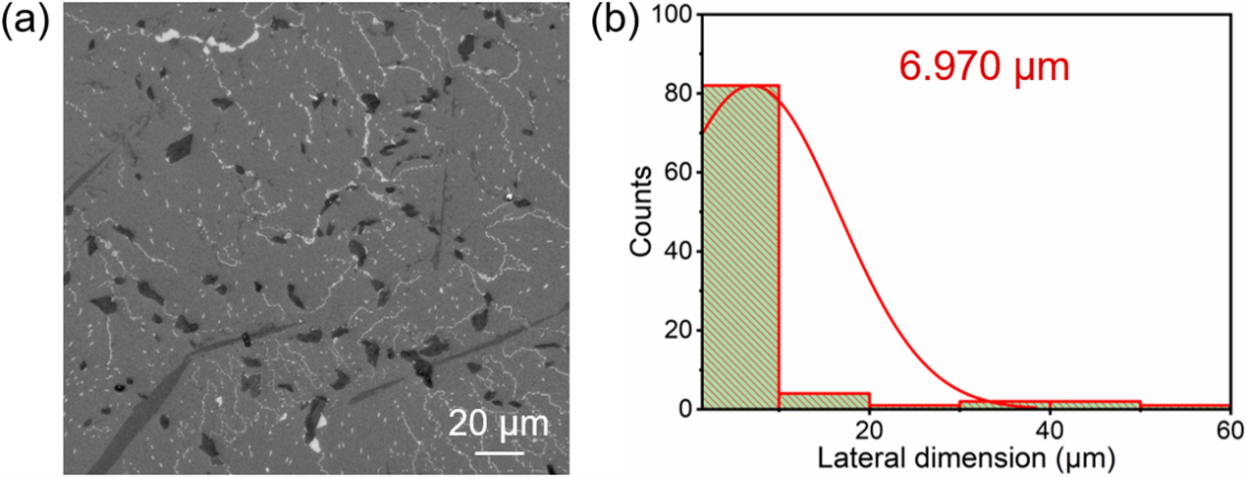


**Figure S7.** (a) The SEM image of bismuth oxyhydroxide nanosheets drop-casted on SiO_2_ substrate and (b) corresponding lateral dimension statistical distribution.


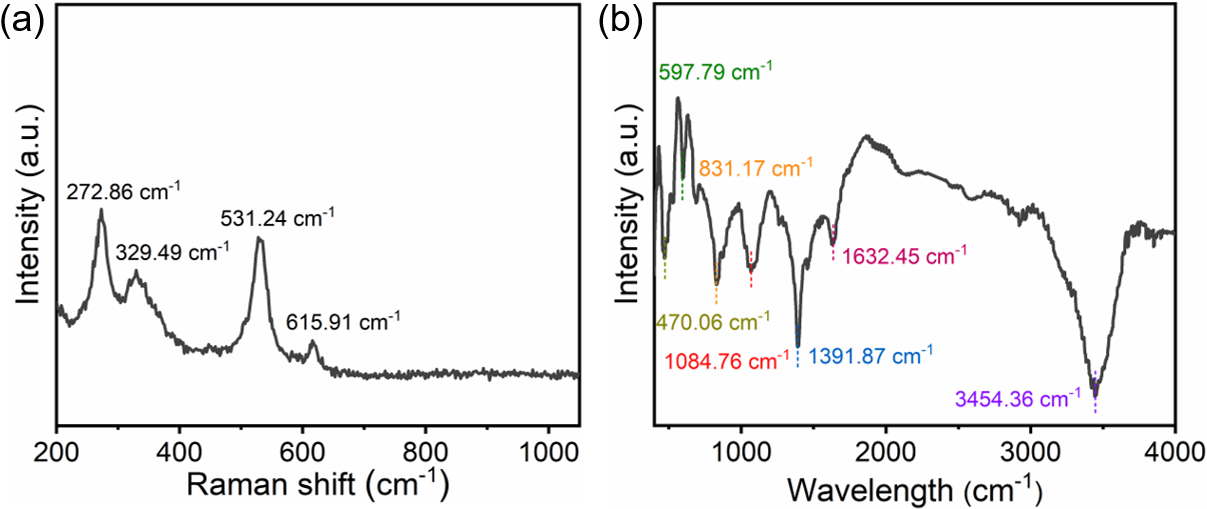


**Figure S8.** a) Raman spectra, and b) FTIR spectra of bismuth oxyhydroxide.


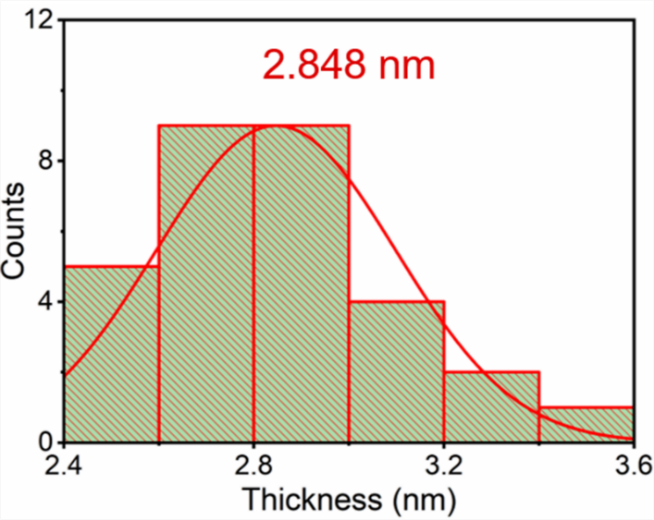


**Figure S9.** The statistical distribution of the thickness of bismuth oxysulfide obtained in 0.1 mol/L Na_2_S solution.


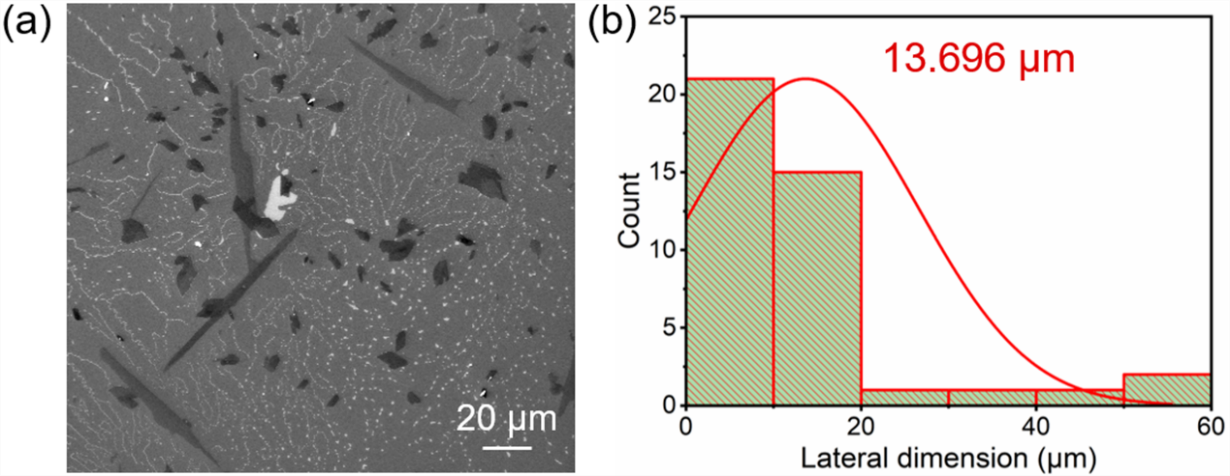


**Figure S10.** a) The SEM image of bismuth oxysulfide obtained in 0.1 mol/L Na_2_S solution, and b) corresponding lateral dimension statistical distribution.


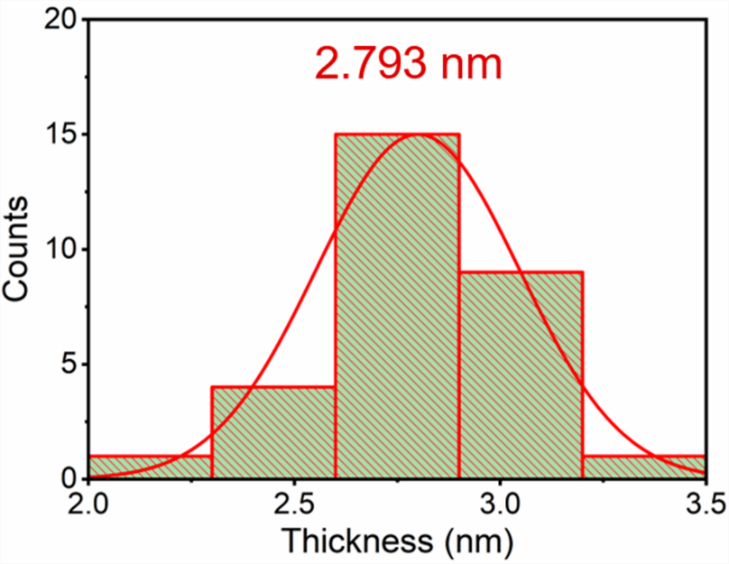


**Figure S11.** The statistical distribution of the thickness of bismuth oxysulfide obtained in 1 mol/L Na_2_S solution.


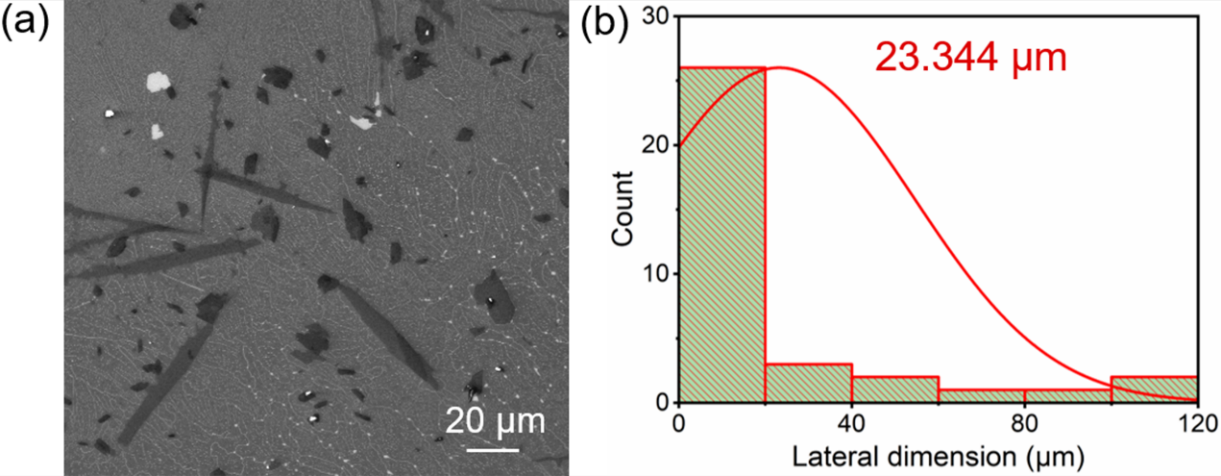


**Figure S12.** a) The SEM image of bismuth oxysulfide obtained in 1 mol/L Na_2_S solution, and b) corresponding lateral dimension statistical distribution.


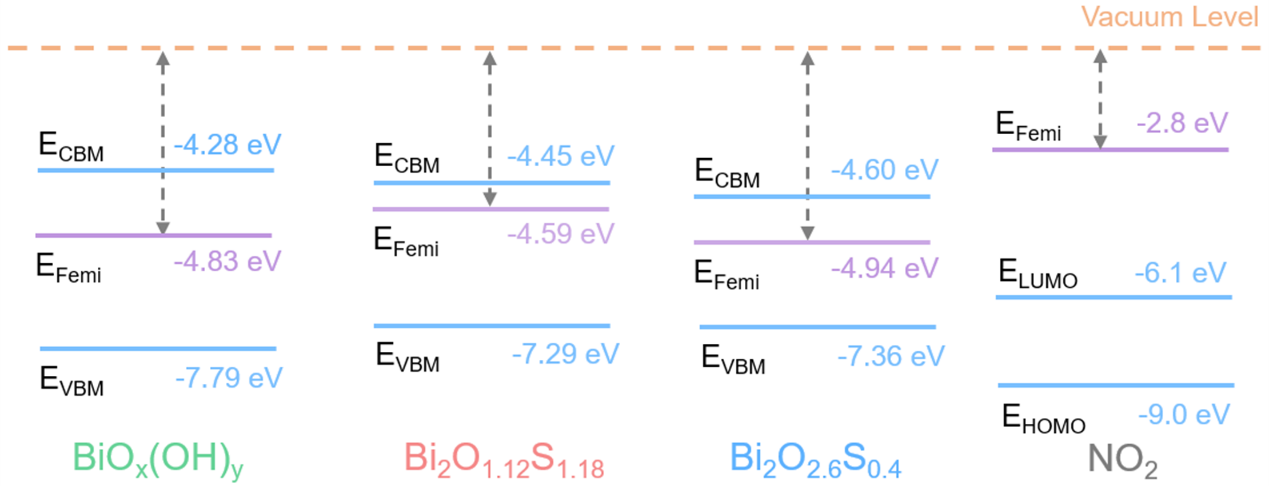


**Figure S13.** Energy band diagram of bismuth oxyhydroxide, bismuth oxysulfide, and NO_2_ molecule.

**Table S1.** The sensing performance comparison of Bi_2_O_1.22_S_1.88_ based sensor towards 100 ppb NO_2_ under the different light irradiation environment.

| Light source | Wavelength  (nm) | Response  (%) | Response time (min) | Recovery time (min) |
| --- | --- | --- | --- | --- |
| UV | 365 | 26.7 | 7.6 | 10.2 |
| Purple | 405 | 149.1 | 8.2 | 11.8 |
| Blue | 450 | 89.8 | 8.8 | 12.1 |
| No light | - | 39.2 | 8.9 | - |


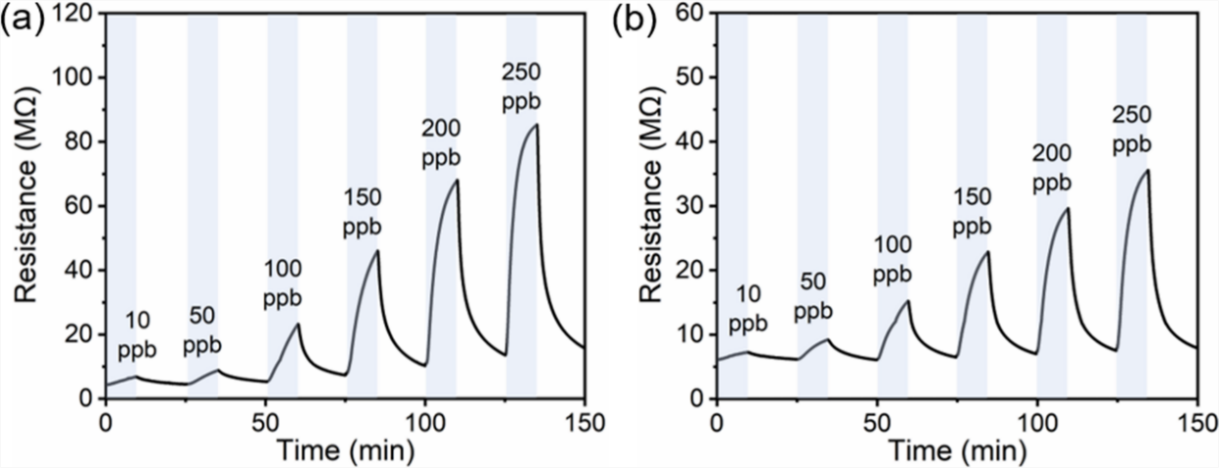


**Figure S14.** The dynamic sensing response of two gas sensors based on Bi_2_O_1.22_S_1.88_ towards NO_2_ under purple light excitation at room temperature.





**Figure S15.** The gas response of the sensor based on Bi_2_O_1.22_S_1.88_ towards 250 ppb NO_2_ at purple light irradiation and different humidity level.
